# Regulation of food intake by Connexin43 via adipocyte-sensory neuron electrical synapses

**DOI:** 10.1016/j.molmet.2025.102247

**Published:** 2025-09-05

**Authors:** Xi Chen, Xing Fang, Hong Zhou, Jieyi Meng, Yang He, Leon G. Straub, Andrew Lemoff, Clair Crewe, Shangang Zhao, Yong Xu, Yi Zhu

**Affiliations:** 1 Children's Nutrition Research Center, Department of Pediatrics, Baylor College of Medicine, Houston, TX, 77030, USA; 2 Jan and Dan Duncan Neurological Research Institute at Texas Children's Hospital, Baylor College of Medicine, Houston, 77030, USA; 3 Department of Biochemistry and Molecular Cell Biology, University Medical Center Hamburg-Eppendorf, Martinistr. 52, 20246, Hamburg, Germany; 4 Department of Biochemistry, The University of Texas Southwestern Medical Center at Dallas, Dallas, TX, 75390, USA; 5 Department of Cell Biology and Physiology, Washington University School of Medicine, St. Louis, MO, USA; 6 Barshop Institute for Longevity and Aging Studies, Division of Endocrinology, Department of Medicine, University of Texas Health Science Center at San Antonio, San Antonio, TX, USA

**Keywords:** Connexin 43, Gap junction, Food intake, Adipocytes, Sensory neurons, Electrical synapses

## Abstract

**Background and objective:**

Connexin43 (Cx43), encoded by *Gja1*, forms gap junctions between adjacent cells. In adipose tissue, it is upregulated during adipose beiging while downregulated by high-fat-diet (HFD) feeding. Adipocyte-specific *Gja1* overexpression enhances adipose tissue beiging in response to mild cold stress of room temperature. Moreover, those mice display a surprising decrease in food intake, but the mechanism remains unclear. This study investigates how adipocyte Cx43 influences feeding behavior.

**Methods:**

Mice with adipose tissue-specific *Gja1* overexpression (Adipoq-Cx43) were fed with HFD. Food intake, weight gain, substrate utilization, and serum lipolysis were assessed. RNA-seq, proteomics, and cytokine measurements were employed to identify candidate signals. Sensory neurons were manipulated via subcutaneous capsaicin injection or iWAT-targeted optogenetics. Co-culture of adipocytes and sensory neurons in vitro was used to test gap junction communication between these two types of cells.

**Results:**

Adipoq-Cx43 mice showed reduced food intake, fat mass, and weight gain on HFD, and shifted substrate utilization toward fatty acids. Although GDF15 was elevated, its neutralization did not reverse the reduced food intake. Instead, systemic ablation of sensory neurons using capsaicin abolished the suppressed food intake. Ooptogenetic activation of sensory neurons in iWAT acutely reduced food intake and improved glucose tolerance after two weeks. In the co-culture of adipocytes and in vitro differentiated sensory neurons, optogenetic stimulation of adipocytes enhanced firing of the adjacent sensory neurons via gap junctions, an effect blocked by the gap junction inhibitor carbenoxolone.

**Conclusions:**

Gap junction–mediated electrical communication between adipocytes and sensory neurons may regulate feeding.

## Highlights

• Adipocyte Cx43 overexpression reduces food intake and weight gain in mice on a high-fat diet.
• Classical adipokines (e.g., leptin, GDF15) are dispensable for this pathway.
• Optogenetic activation of inguinal WAT sensory neurons mimics Cx43's effects, while sensory neuron ablation abolishes them.
• Co-culture experiments confirm adipocytes directly modulate sensory neuron activity via gap junctions.
• Gap junctions between adipocytes and sensory neurons may mediate appetite suppression via electrical signaling in vivo.

## 1 Introduction

While serving as the primary fat storage, white adipose tissue (WAT) is actively regulated by the central nervous system and blood-transported hormones [1,2]. WAT also communicates with other organs via secreted metabolites, adipokines, and nerves to regulate food intake and energy metabolism [[3], [4], [5], [6], [7]].

Within the tissue, WAT is not just a pile of adipocytes. In contrast, they are separated by connective tissue septa and organized in patches [8] and also supported by blood vessels and innervated by nerve fibers [9], although at different densities in the lean and obese states. Immune cells infiltrate the tissue via blood and lymphatic vessels, especially under an obese state, to participate in the inflammatory process and subsequently affect the adipocyte metabolism and WAT tissue function. Many mechanisms are utilized to communicate among adipocytes and other types of cells, e.g., gap junctions, secreted proteins, neural signaling, and exosomes [10]. We previously found that adipocyte Cx43 gap junctions play an important role in disseminating sympathetic beiging signals and that overexpressing *Gja1* increases adipocyte *Ucp1* abundance–a key beige adipocyte marker–under room temperature [11]. Later, we found that the efficacy of blood-borne adipose tissue–acting molecules can also be further enhanced by a Cx43 gap junction channel activator, danegaptide, offering a new route to improve the effectiveness of those adipose tissue–targeting pharmaceutical treatments [12].

WAT of diet-induced obese mice has reduced intercellular gap junctions, and deletion of *Gja1* in adipocytes impaired the WAT's adaptation to cold or lactation [11,13]. In this report, we show that overexpression of Cx43 exerts metabolic benefits and a surprising reduction in food intake. In search of how adipocyte-specific overexpressing Cx43 mediates this high-level central-controlled behavior, we found that sensory neurons are implicated in the process after excluding the role of adipocyte-released FFA, leptin, or GDF15. Then, using wireless optogenetics in mice and a co-culture of adipocytes and differentiated sensory neurons, we found that adipocytes can communicate with sensory neurons via gap junctions to mediate sensory neurons' electrical firing. Moreover, activating sensory neurons in the WAT depot is sufficient to suppress food intake. Based on these data, we propose that the adipocyte state may be monitored by sensory neurons via gap junctions between adipocytes and sensory neurons, a type of electrical synapse, to regulate food intake.

## 2 Materials and Methods

### 2.1 Animals

TRE*-Gja1* mice were generated and characterized previously [11]. The established founder was crossed to Adipoq-rtTA mice (Jax #033448) [14] to generate Adipoq-rtTA::TRE-*Gja1* double transgenic mice, also referred to as Adipoq-Cx43. Those mice are on pure C57BL/6J background. To generate Advillin-Cre:LSL-ChR2 double transgenic mice (referred to as AChR mice), Advillin-Cre (Jax #032536) mice were crossed with Rosa-CAG-LSL-ChR2(H134R)-EYFP-WPRE mice (abbreviated as LSL-ChR2, Jax #024109). Similarly, TH1-Cre mice (Jax #008601) were crossed with LSL-ChR2 mice to generate TH1-Cre:LSL-ChR2 double transgenic mice, referred to as TChR mice. Adipoq-Cre (Jax #028020) mice were crossed with LSL-ChR2 mice to generate Adipoq-Cre:LSL-ChR2 double transgenic mice, referred to as Adipoq-ChR mice. Those mice are on a mixed background. All animals were kept on a 12-hour light–dark cycle in a temperature-controlled environment. Mice were free to access water and fed one of the following: a standard chow diet (LFD), a 60% high-fat diet (HFD; BioServ, S1850), or a 60% HFD containing 200 mg/kg doxycycline (BioServ, S6223). Mice were genotyped by Transnetyx. Animal care and experimental protocols were approved by the Institutional Animal Care and Use Committee of the Baylor College of Medicine.

### 2.2 RNA extraction and qRT-PCR

RNA was isolated from frozen tissues by homogenization in Trizol Reagent (Invitrogen, 15596018) as previously described [15]. RNA concentrations were quantified using a NanoDrop Spectrophotometer; cDNA was reverse transcribed from 1 μg of RNA using a reverse transcription kit (Bio-Rad), and qRT-PCR primers were obtained from the Harvard PrimerBank [16] and are listed in Supplementary Table 1. The messenger RNA levels were calculated using the comparative threshold cycle method, normalized to gene Rps16.

### 2.3 RNA sequencing

RNA was isolated as described in 2.2, and RNA integrity was verified using an Agilent 2100 Bioanalyzer (Agilent Technologies). Only samples with RIN values above 8.0 were used for experiments; cDNA libraries were prepared by using an Illumina TruSeq RNA sample prep kit. The average size of the libraries cDNAs was 150 bp (excluding adapters). The integrity and quality of cDNA libraries were assessed using an Agilent 2100 Bioanalyzer and an ABI StepOne Plus real-time PCR system. RNA-seq was performed by Novogene. Raw read sequencing quality and adapter contamination were assessed using FastQC v0.11.9. Overall quality was determined to be satisfactory, and raw reads were aligned to the mouse genome index using STAR v2.7.9a. The STAR genome index was created using raw FASTA and annotation files downloaded from the GENCODE portal for mouse genome build GRCm38 release 23. Alignments were saved in binary format (BAM). Summary of raw read quality and alignment quality were generated using MultiQC v1.12 [17]. Sample-specific gene expression values were computed as the number of reads aligned per gene using STAR –quantMode GeneCounts. Raw counts were normalized, and genes with an average read count <50 across all samples were considered unexpressed and excluded from the differential analysis. The analysis for differential gene expression was carried out using DESeq2 [18]. A false discovery rate (FDR) cutoff of 0.05 and a fold change cutoff of 20% (−0.263 ≤ log2(FC) ≥ +0.263) were imposed to identify significant differentially expressed genes. Genes identified in different contrasts were then overlapped to determine directionality of differential gene expression between contrasts.

### 2.4 Western blotting

Protein extraction and abundance determination were performed as previously described [19]. Specific protein targets were detected using primary antibodies Connexin 43 (Abclonal, A23120; 1:1000 dilution), α-Tubulin (CST, 3873T; 1:5000 dilution), HSL (Santa Cruz, sc-74489; 1:500 dilution) and phosphor-HSL (Ser565; CST, 4137T; 1:1,000 dilution). Secondary antibodies used include Goat anti-Mouse Alexa Fluor® Plus 800 (Invitrogen, A32730) and Goat anti-Rabbit Alexa Fluor® Plus 680 (Invitrogen, A32734), both at 1:10,000 dilutions. Antibody-decorated membranes were then visualized on a Li-Cor Odyssey infrared scanner, and the scanned data were analyzed using Odyssey version 3.0 software.

### 2.5 Histology

After the mice were euthanized, the tissues were excised immediately, fixed overnight in 10% PBS-buffered formalin, and stored in 50% ethanol. Tissues were sectioned (5 μm), rehydrated, and stained with hematoxylin and eosin (H&E) at the Pathology Core at BCM. Microscopic images were taken on a ZEISS Axioscan scanner.

### 2.6 Glucose tolerance test

Glucose tolerance tests were performed as described previously [21]. In brief, the mice fasted for 4–6 h, after which glucose solution (10 μl/g body weight) was administered orally or by intraperitoneal injection; the final dose of glucose was 1.25 g/kg body weight for the mice on LFD and 0.75 g/kg body weight for the mice on HFD. The lower glucose dose for mice on HFD prevents blood glucose from reading over the detection limit of the glucometer used. Blood glucose levels before and 15, 30, 60, and 120 min after the glucose injection were measured using a glucometer.

### 2.7 Serum lipids

Serum triglycerides were measured using Infinity reagent. Serum non-esterified free fatty acids were measured using NEFA-HR assay (Wako, C1057). Total cholesterol and HDL cholesterol were measured using an AF HDL and LDL/VLDL assay kit (Sigma–Aldrich, MAK331). Measurements were carried out using manufacturer-provided protocols.

### 2.8 Glycerol release assay

Epididymal fat pads were surgically removed from male mice and washed with ice-cold PBS. Fat pads (about 30–50 mg) were preincubated for 1 h in 250 μL of DMEM (Life Technologies, 10569044) containing 2% fatty acid–free BSA (Fisher Scientific, BP9704100). Subsequently, fat pads were cut into several pieces and incubated in 250 μL of KRH buffer (125 mM NaCl; 5 mM KCl; 1.8 mM CaCl2; 2.6 mM MgSO4; 5 mM HEPES; pH 7.2) plus 2% BSA (fatty-acid free) with or without the presence of 5 μM CL 316,243 (Sigma, C5976) for 2 h at 37 °C. Free glycerol content was quantified for each sample in the medium using the Free-Glycerol Determination Kit (Sigma, F6428-40 ML). Glycerol release from each sample was normalized to the weight of each fat pad.

### 2.9 CL 316,243-stimulated glycerol release

Mice were fasted for 4 h prior to the experiment. The β3-adrenergic receptor agonist CL 316,243 (Sigma, C5976) was administered via intraperitoneal injection at a dose of 1 mg/kg body weight, as previously described [20]. Blood samples were collected at 0 min as well as 5, 15, and 30 min post injection. Glycerol content in the supernatant was quantified using a free glycerol determination kit (Sigma, F6428-40 ML) according to the manufacturer's instructions.

### 2.10 Metabolic cage

Metabolic cage studies were conducted using a PhenoMaster System (TSE systems). Mice were acclimated in temporary holding cages for 3 days before recording. Food intake, movement, and CO_2_ and O_2_ levels were measured at various intervals (determined by collectively how many cages were running concurrently) for the indicated period shown in the figures.

### 2.11 Subcutaneous thermal probe implantation and body temperature measurement

To monitor body temperature during cold exposure experiments, an IPTT-300 transponder (Bio Medic Data Systems) was subcutaneously implanted in mice under isoflurane anesthesia at least 1 week before the experiments. The transponder was inserted using a presterilized 12-gauge disposable needle in a syringe-like manner, positioning it far from the brown adipose tissue (BAT) on the dorsal side. Subcutaneous temperature was recorded using the DAS-8027IUS wireless reader (Bio Medic Data Systems). For acute cold exposure experiments, mice were transferred to a cold room maintained at 4 °C and were singly housed in cages with Enviropaks for up to 6 h, with no access to food. Core body temperature was recorded every 30 min from freely moving animals using the DAS-8027IUS wireless reader.

### 2.12 Serum FGF21, GDF15, leptin, and cytokine measurement

Blood samples were collected using EDTA-coated tubes and centrifuged at 2,000 g for 20 min within 30 min of collection. The separated serum was aliquoted and stored at −20 °C until analysis. Serum leptin, FGF21, and GDF15 were measured using ELISA. Leptin was quantified using the Mouse/Rat Leptin Quantikine ELISA Kit (R&D Systems), FGF21 using the Mouse/Rat FGF-21 Quantikine ELISA Kit (R&D Systems), and GDF15 using the Mouse/Rat GDF-15 Quantikine ELISA Kit (R&D Systems)—all following the manufacturer's instructions. Cytokines were measured at Eve Technologies (Calgary, Canada).

### 2.13 Serum proteomics

Major serum proteins—including albumin, α-antitrypsin, transferrin, and haptoglobin—were deleted from serum using ProteoSpin™ Abundant Serum Protein Depletion (Norgen Biotek, 17300). The depleted serum was loaded onto the top of an SDS-PAGE gel, run approximately 1 cm into the gel, and then stained with Coomassie Blue. After destaining, the protein-containing region of the gel was excised, diced into cubes, and placed into 1.5 mL conical-bottom centrifuge tubes. Gel pieces were reduced with DTT (20 mM) and alkylated with iodoacetamide (27.5 mM). A 0.01 μg/μL solution of trypsin in 50 mM triethylammonium bicarbonate (TEAB) was added to completely cover the gel pieces, which were subsequently incubated on ice. Then, 50 μL of 50 mM TEAB was added, and the gel pieces were digested overnight (Pierce). Following solid-phase extraction cleanup with an Oasis HLB elution plate (Waters), the resulting peptides were reconstituted in 10 uL of 2% (v/v) acetonitrile (ACN) and 0.1% trifluoroacetic acid in water. A 5 uL aliquot was injected onto an Orbitrap Fusion Lumos mass spectrometer (Thermo Electron) coupled to an Ultimate 3000 RSLC-Nano liquid chromatography system (Dionex). Samples were loaded onto a 75-μm inner diameter, 50-cm long EasySpray column (Thermo) and eluted using a gradient of 1–28% buffer B over 60 min. Buffer A contained 2% (v/v) ACN and 0.1% formic acid in water, and buffer B contained 80% (v/v) ACN, 10% (v/v) trifluoroethanol, and 0.1% formic acid in water. The mass spectrometer operated in positive ion mode with a source voltage of 2.4 kV and an ion transfer tube temperature of 275 °C. MS scans were acquired at a resolution of 120,000 in the Orbitrap, and up to 10 MS/MS spectra were obtained in the ion trap for each full spectrum acquired using higher-energy collisional dissociation (HCD) for ions with charges 2–7. Dynamic exclusion was set for 25 s after each ion was selected for fragmentation.

Raw MS data files were analyzed using Proteome Discoverer v2.2 (Thermo), with peptide identification performed using Sequest HT searching against the Uniprot mouse protein database. Fragment and precursor mass tolerances of 0.6 Da and 10 ppm were specified, respectively, and 3 missed cleavages were allowed. Carbamidomethylation of Cysteine was specified as a fixed modification, and oxidation of Methionine was specified as a variable modification. The false discovery rate was set to 1% for all peptides. All proteomics raw data and peak list files were uploaded to the MassIVE data repository, with dataset accession number MSV000097803.

### 2.14 Co-culture of SVF and F11 cells

The F11 cell line was obtained from ATCC and cultured in Dulbecco's Modified Eagle Medium (DMEM) supplemented with 10% fetal bovine serum (FBS) and 100 U/mL penicillin-streptomycin (P/S). A stable F11 mScarlet3-expressing cell pool was generated by transfecting F11 cells with a pSB-EF1aHT-mScarlet3-H plasmid, which was constructed by inserting the mScarlet3 coding sequence downstream of the EF1aHT promoter in a Sleeping Beauty transposon backbone, followed by selection with 250 μg/mL hygromycin for 14 days. Cells of passages 6–8 were used for all experiments.

Primary stromal vascular fraction (SVF) cells were isolated from Ad-ChR2 mice as previously described [21] and seeded onto coverslips placed in 12-well plates. The SVF was cultured in DMEM/F-12 medium (ThermoFisher, 11330057) containing 10% FBS (ThermoFisher, 16140071), penicillin (100 UI/mL), streptomycin (100 μg/mL; Sigma Aldrich, P4333-100 mL), and 0.1% gentamycin (ThermoFisher, 15710064). The culture medium was refreshed every other day until the cells reached 100% confluence. To induce differentiation into mature adipocytes, a differentiation cocktail consisting of 5 μg/mL insulin (Thomas Scientific, C979A70), 1 μM dexamethasone (Sigma Aldrich, D4902-25 MG), 0.5 mM isobutylmethylxanthine (Sigma Aldrich, 15879), and 1 μM rosiglitazone (Sigma Aldrich, R2408-10 MG) was added to the medium for 3 days. On Day 4, the medium was replaced with only DMEM/F-12 containing 5 μg/mL insulin to maintain differentiation, and F11 Scarlet cells were co-cultured with SVF cells at a 1:200 ratio. On Day 5, the medium was switched to a co-differentiation medium composed of DMEM/F-12 supplemented with 3–5% FBS, 5 μg/mL insulin, 50 ng/mL nerve growth factor (NGF; Novus ordered through VWR,556-NG-100/CF), 0.5 mM dibutyryl-cAMP (Sigma Aldrich, D0260-5 mg), 0.5% Insulin-transferrin-sodium selenite (Fisher Scientific, 41-400-045), and 2 μM retinoic acid (Benchmark Scientific, D1134-50). This medium was refreshed every other day for 5–6 days. Fully differentiated adipocytes and F11 neurons were subsequently used for electrophysiological studies.

### 2.15 Electrophysiology

F11 cells on their fifth and sixth differentiation days were used for experiments, with passages ranging from 6 to 8. Healthy F11s with sufficiently large round somas were selected for the study. F11-mScarlet3 cells that were not in direct contact with mature adipocytes and such cells that were in direct contact with mature adipocytes were both used in the experiments for comparison.

For electrophysiological recordings, the culture medium was replaced with a standard artificial cerebrospinal fluid (aCSF) containing 126 mM NaCl, 2.5 mM KCl, 2.4 mM CaCl_2_, 1.2 mM NaH_2_PO_4_, 1.2 mM MgCl_2_, 11.1 mM glucose, and 21.4 mM NaHCO_3_ (pH 7.4). The aCSF was maintained at 30 °C and continuously bubbled with a gas mixture of 95% O_2_ and 5% CO_2_. Patch pipettes with resistances of 4–7 MΩ were filled with intracellular solution (pH 7.3) containing 128 mM K-gluconate, 10 mM KCl, 10 mM HEPES, 0.1 mM EGTA, 2 mM MgCl_2_, 0.05 mM Na-GTP, and 4 mM Mg-ATP. Recordings were performed using a MultiClamp 700B amplifier (Axon Instrument), digitized via a Digidata 1440A interface, and analyzed offline with pClamp v10.3 software (Axon Instruments). Additionally, mScarlet3-labeled neurons were visualized using epifluorescence and IR-DIC imaging on an upright microscope (BX51WI, Olympus) equipped with a motorized stage (MP-285, Sutter Instrument). Series resistance was monitored during the recording: typically <10 MΩ and not compensated. The liquid junction potential was corrected after the experiment. Data were excluded if the series resistance increased dramatically during the experiment or without overshot for the action potential. Currents were amplified, filtered at 1 kHz, and digitized at 20 kHz. The current clamp was engaged to test neural firing frequency and resting membrane potential at the baseline or in response to blue light stimulation (20 Hz). A baseline spontaneous firing period was recorded for 20 s, followed by 40 s of blue light stimulation and a final 20-second post-stimulation recording. For CBX experiments, the same recording procedure was performed twice: once under control conditions and again after incubating the slices with 1 mM CBX in aCSF for 3 min.

### 2.16 Optogenetic probe implantation and light stimulation

At 10 weeks of age, mice underwent the implantation of a Neurolux 470 nm spinal device (Neurolux, Urbana, IL) into the iWAT on one side. Briefly, mice were anesthetized using isoflurane/oxygen, and a vertical incision (1–1.5 cm) was made on the lower back, approximately 0.5 cm lateral to the midline. Blunt-ended forceps were used to carefully separate the skin from the underlying iWAT, taking care to avoid damaging the innervating nerves. Once an adequate portion of the iWAT was exposed, 1% toluidine blue was used to indicate nerves. Nerve fibers coming from the skin to the iWAT (usually 3) were cut to avoid unspecific stimulation of skin sensory nerves that travel through the iWAT. The optogenetic probe was positioned to cover the dorsal part of the iWAT. The incision was then sutured securely. Following the surgery, mice were allowed to recover for at least 2 weeks before optogenetic stimulation.

After recovering from surgery, the mice were individually housed in wireless optogenetic cages. The mice were fasted overnight and then refed the following morning for 1 h to collect food intake with or without blue light photostimulation (470 nm, 10 W, 10 Hz, 10% duty cycle) in a specialized wired cage during the refeeding period.

### 2.17 Statistical analysis

Results are shown as mean ± SEM. For experiments with two groups, Student's *t*-test was utilized. For studies with three or more groups, one-way ANOVA was used; for experiments with several groups with a balanced distribution of two factors, two-way ANOVA test was used. A Sidak test was used for post-hoc analysis of comparisons within subgroups. Also, *p* values < 0.05 were considered statistically significant. Additional details are provided in the figure legends.

## 3 Results

### 3.1 Inducible adipocyte Cx43 overexpression suppresses food intake and reduces weight gain in mice

**Figure 1:**
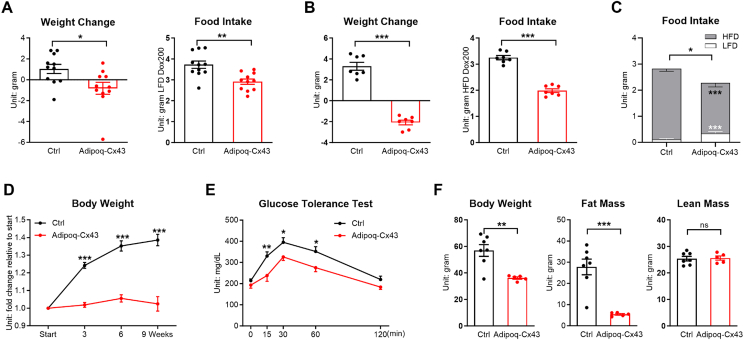
Inducible adipocyte Cx43 overexpression suppresses food intake and reduces weight gain. (A) Weight change and food intake of control and Adipoq-Cx43 mice treated with Dox200 LFD for 1 week (n = 11 mice per group). (B) Weight change and food intake of control and Adipoq-Cx43 mice treated with Dox200 HFD for 1 week (n = 7 mice per group). (C) Food intake of control and Adipoq-Cx43 mice treated with both Dox200 LFD and Dox200 HFD food (n = 11 mice per group). (D) Body weight of control and Adipoq-Cx43 mice treated with Dox200 HFD for 9 weeks (n = 7 mice per group). (E) Glucose tolerance test of control and Adipoq-Cx43 mice treated with Dox200 HFD at Week 3 (n = 7 mice per group). (F) Body weight, fat mass, and lean mass of control and Adipoq-Cx43 mice treated with 10 weeks of Dox200 HFD (n = 7 mice per group). For Panels (A)–(C) and (F), comparisons between genotypes were performed using unpaired two-tailed *t*-tests; in Panels (D) and (E), time point–specific comparisons between genotypes were analyzed using serial *t*-tests. All data are mean ± SEM. ∗∗∗*P* < 0.001, ∗∗*P* < 0.01, and ∗*P* < 0.05.

TRE-Cx43 mice were previously generated and verified [11]. Gene overexpression was induced in Adipoq-rtTA::TRE-Cx43 (referred to as Adipoq-Cx43) mice by supplementing mice with 200 mg/kg doxycycline (Dox) in the diet. The overexpression of the transgene and induction of Cx43 protein expression in adipose tissue were confirmed by qRT-PCR and Western blotting (Fig. S1). One week after dox-containing low-fat diet (Dox200 LFD) treatment, Adipoq-Cx43 mice lost 0.82 g of body weight in comparison to 1.04 g gain of body weight in Adipoq-rtTA control mice, and they consumed 21.8% less dox chow food (Figure 1A). When mice were treated with Dox200 HFD, the weight difference and food intake difference were even greater (Figure 1B). Interestingly, when presented with both Dox200 LFD and Dox200 HFD food, the control mice exclusively chose Dox200 HFD; in contrast, Adipoq-Cx43 mice would eat relatively much more Dox200 LFD, despite a suppression of overall food intake (Figure 1C). When Adipoq-Cx43 mice were chronically treated with Dox200 HFD, they barely gained any weight in contrast to control mice (Figure 1D), and glucose tolerance was significantly improved as early as 3 weeks after the dietary treatment (Figure 1E). After 10 weeks of Dox HFD challenge, QMR analysis showed that lean mass was preserved in Adipoq-Cx43 mice, and the difference in body weight was primarily coming from the fat mass (Figure 1F). These results indicate that adipose tissue Cx43 overexpression could suppress food intake and reduce body weight gain.

### 3.2 Adipoq-Cx43 mice quickly shift to fat metabolism and lower energy expenditure, but mice are still more resistant to cold challenges

**Figure 2:**
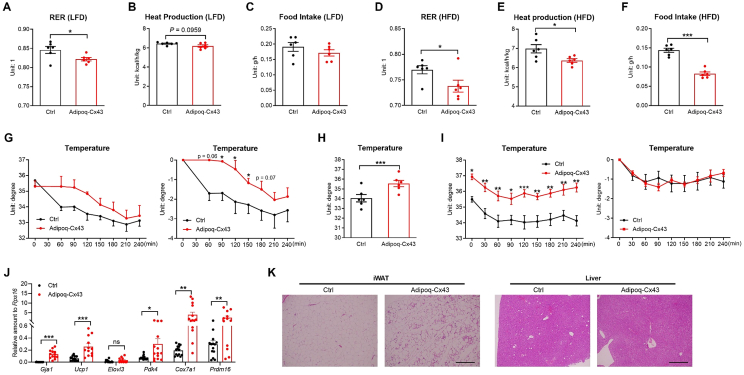
Adipoq-Cx43 mice quickly shift to fat metabolism and lower energy expenditure, but mice are still more resistant to cold challenges. (A) RER, (B) heat production, and (C) food intake of control and Adipoq-Cx43 mice treated with Dox200 LFD (n = 6 mice per group). (D) RER, (E) heat production, and (F) food intake of control and Adipoq-Cx43 mice treated with Dox200 HFD (n = 6 mice per group). For Panels (A)–(F), data from the first 12 h were excluded from the bar graphs and subsequent analyses due to the time required for the TRE system to induce Cx43 gene expression. (G) The body temperature (left) and the change from baseline (right) of control and Adipoq-Cx43 mice after 7-day Dox200 HFD during acute cold exposure at 6 °C (n = 6 mice for control; n = 3 mice for Adipoq-Cx43). (H) The body temperature of control and Adipoq-Cx43 mice on Dox200 HFD after 3-week chronic cold exposure at 12 °C. (I) The body temperature (left) and the change from baseline (right) of control and Adipoq-Cx43 mice on Dox200 HFD during acute cold at 6 °C after 3-week 12 °C exposure followed by 4-hour recovery at RT (n = 6 mice per group for Panels (H) and (I)). For panel (G)–(I), the subcutaneous temperature was measured in lower dorsolumbar region, adjacent to the hindlimb base, by implanting a probe beneath the skin of the mouse on the back oneweek before the experiment. (J) Expression of genes involved in the beiging process in iWAT from control and Adipoq-Cx43 mice after 3-week cold exposure on Dox200 HFD (n = 12 or 14 mice per group). (K) Representative histology of iWAT and liver from control and Adipoq-Cx43 mice after 3-week cold exposure on Dox200 HFD. Scale bar = 50 μm for iWAT and 200 μm for liver. For Panels (A)–(F), (H), and (J), comparisons between genotypes were performed using unpaired two-tailed *t*-tests; in Panels (G) and (I), time point–specific comparisons between genotypes were analyzed using serial *t*-tests. All data are mean ± SEM. ∗∗∗*P* < 0.001, ∗∗*P* < 0.01, and ∗*P* < 0.05.

To further characterize the Adipoq-Cx43 mice, we exposed them to metabolic cages. On Dox200 LFD, Adipoq-Cx43 mice quickly developed a lower respiratory exchange ratio (RER; Figure 2A and Fig. S2A) presumably after transgene overexpression, indicating a switch from glucose to fat oxidation, which is accompanied by lower heat production calculated from oxygen consumption (Figure 2B and Fig. S2B). It is worth noting that in the cage setting, the mice have not shown a statistically significant reduction in food intake (Figure 2C and Fig. S2C), probably due to the short period of transgene overexpression and changes in housing conditions that masked a would-be small change in food intake on LFD. However, the curves do diverge after 1 day. Water intake and activities were not different in Adipoq-Cx43 mice when compared to Adipoq-rtTA controls (Fig. S2D and S2E). Adipoq-Cx43 mice lost fat mass and gained lean mass as a percentage of body weight, while the body weight was weakly trending lower (Fig. S2F).

In contrast, on Dox200 HFD, Adipoq-rtTA mice had a lower RER than that on LFD, as expected for mice on a lipid-enriched diet, and Adipoq-Cx43 mice showed an even stronger decrease in RER (Figure 2D and Fig. S2G). Adipoq-Cx43 also showed a reduction in heat production (Figure 2E and Fig. S2H). Intake of Dox200 HFD was significantly reduced (Figure 2F and Fig. S2I). This coexists with increased Z movement (Fig. S2J), which usually reflects food-seeking behavior, probably reflecting a decreased hedonic value of HFD. Water intake and X and Y movements were not different (Fig. S2K, S2L). Unlike on Dox200 LFD, Adipoq-Cx43 mice on Dox200 HFD have a significant decrease in body weight during this period, when losing fat mass and gaining lean mass in percentage (Fig. S2M). These data suggest that Adipoq-Cx43 mice quickly shift to fat metabolism on Dox200 HFD.

Despite a reduction in heat production at room temperature (RT), Adipoq-Cx43 mice were more cold-tolerant on Dox200 LFD (Figure 2G). In another experimental cohort, Adipoq-Cx43 had a higher body temperature after a chronic 3-week cold challenge at 12 °C on Dox200 HFD (Figure. 2H). Adipoq-Cx43 mice maintained higher body temperature during the cold challenge (6 °C) after being moved to RT for 4 h on Dox200 HFD (Figure 2I). Adipoq-Cx43 also showed enhanced expression of *Gja1* and beiging genes (*Ucp1*, *Elovl3*, *Pdk4*, *Cox7a1,* and *Prdm16*) in their iWAT after cold exposure, more beige adipocytes, and improved hepatic lipid accumulation (Figure 2J, K). These results indicate that Adipoq-Cx43 mice were more resistant to cold challenge.

### 3.3 Inducible adipocyte-specific Cx43 expression does not promote lipolysis

**Figure 3:**
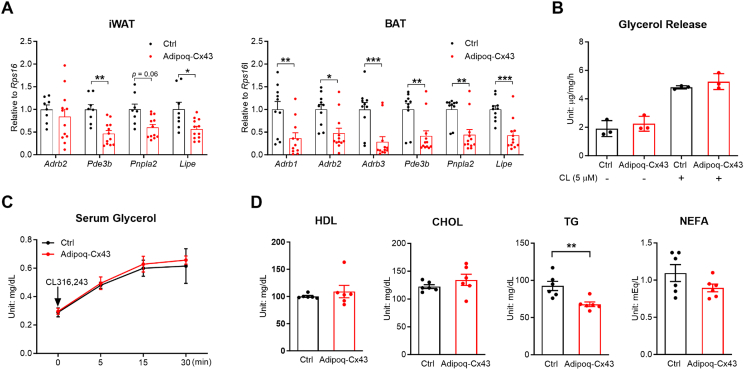
Inducible adipocyte-specific Cx43 expression does not promote WAT lipolysis. (A) Expression of lipid metabolic–related genes in iWAT and BAT from control and Adipoq-Cx43 mice treated with 7 days of Dox200 HFD (iWAT: n = 8 mice for control, n = 12 mice for Adipoq-Cx43; BAT: n = 10 mice for control, n = 12 mice for Adipoq-Cx43; one data point for *Adrb1* in BAT from Adipoq-Cx43 was excluded as an outlier). (B) CL316,243-stimulated glycerol release from the freshly dissected fat pads from control and Adipoq-Cx43 mice treated with Dox200 HFD for 7 days (n = 3 mice per group). (C) Serum glycerol of control and Adipoq-Cx43 mice treated with in vivo CL316,243 stimulation at the indicated time points (n = 5 mice per group; one control group sample at 5 min was not detected due to technical error). (D) Serum lipid levels of control and Adipoq-Cx43 mice after acute Cx43 induction (n = 6 mice per group). HDL: high-density lipoprotein; CHOL: cholesterol; TG: triglycerides; NEFA: non-esterified fatty acids. For Panels (A) and (D), comparisons between genotypes were performed by using unpaired two-tailed *t*-tests; in Panel (D), time point–specific comparisons between genotypes were analyzed using serial *t*-tests; two-way ANOVA was used for Panel (B). All data are mean ± SEM. ∗∗∗*P* < 0.001, ∗∗*P* < 0.01, and ∗*P* < 0.05.

Free fatty acids (FFAs) can suppress appetite by slowing gastric emptying, promoting the secretion of anorectic gut hormones, which subsequently regulate appetite-modulating neurons [22,23]. Thus, we tested whether the adipocyte-specific Cx43 expression promotes lipolysis as the possible mechanism of appetite suppression. Opposite to what we expected, the mRNA expression of Adipoq-Cx43 iWAT and BAT showed a reduction in lipid metabolic–related genes (Figure 3A). The freshly dissected Adipoq-Cx43 fat pad also showed the same level of glycerol release upon stimulation by a β3-adrenergic receptor agonist, CL316,243 (Figure 3B). Adipoq-Cx43 mice did not have a higher glycerol level after in vivo CL316,243 stimulation either (Figure 3C). Fasting serum lipid levels were also largely unchanged, except for a decrease in triglyceride (TG) levels in Adipoq-Cx43 mice (Figure 3D). All these data suggest that adipocyte-specific Cx43 expression does not promote lipolysis.

### 3.4 Search for adipose tissue–secreted proteins that may mediate food intake in Adipoq-Cx43 mice

**Figure 4:**
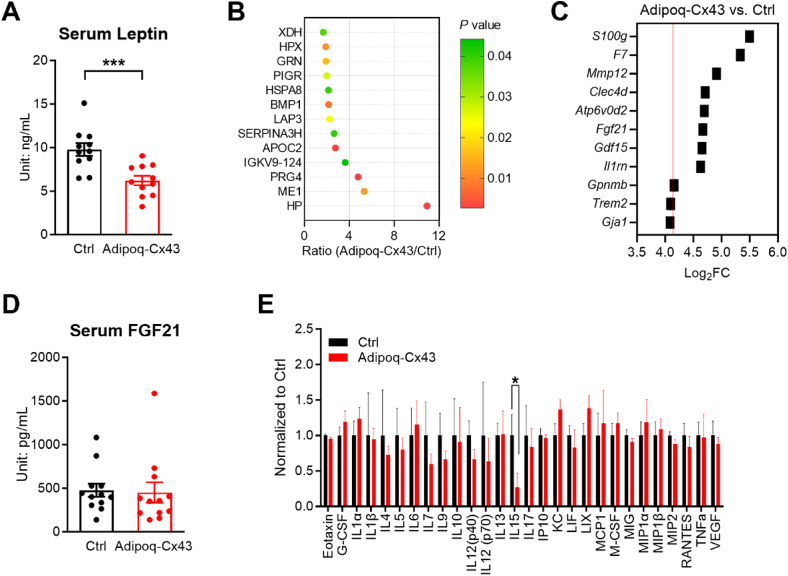
Search for adipose tissue–secreted factors that may mediate food intake in Adipoq-Cx43 mice. (A) Serum leptin for control and Adipoq-Cx43 mice (n = 11 mice per group). (B) Serum proteomics for Adipoq-Cx43 mice. Shown here are proteins that were significantly increased in the Adipoq-Cx43 mice compared to control mice. Horizontal bars represent the ratio (n = 6 mice per group). (C) Genes encoding secreted proteins in the adipose tissue, identified by RNA-seq. Shown here are the top 10 genes significantly upregulated in the Adipoq-Cx43 mice compared to control mice (p < 0.001). Horizontal bars represent the Log2 fold-change comparison (upregulated genes in Adipoq-Cx43 mice were identified on the basis of a threshold log2FC > c0, where c0 represents the Log2 fold-change of *Gja1*). (D) Serum FGF21 for control and Adipoq-Cx43 mice (n = 12 mice per group). (E) The cytokine contents of the serum from control and Adipoq-Cx43 mice (n = 6–8 mice per group). For Panel (C), mice were treated with Dox200 LFD for 1 week. For all other panels, mice were treated with Dox200 HFD for 3 weeks. For Panels (A), (D), and (E), comparisons between genotypes were performed using unpaired two-tailed *t*-tests. All data are mean ± SEM. ∗∗∗P < 0.001, ∗∗P < 0.01, and ∗P < 0.05.

Serum from Adipoq-Cx43 mice showed lower leptin levels compared to Adipoq-rtTA control mice (Figure 4A), which can't explain the phenotype of suppressed food intake, as serum leptin levels are expected to negatively regulate food intake [24,25]. Then, we performed proteomics on serum from Adipoq-rtTA and Adipoq-Cx43 mice that were depleted of the most abundant proteins. The comparison showed 13 proteins that were significantly increased in Adipoq-Cx43 serum (fold change >1.67, p < 0.05; Figure 4B). However, a literature review did not support further investigation of any proteins from the list. Then, we performed RNA-seq to identify upregulated genes in the adipose tissue that encode secreted proteins. Of the top 10 in the upregulated genes, *Fgf21* and *Gdf15* encode secreted hormones (Figure 4C). Despite that, there was no difference in serum FGF21 levels between Adipoq-rtTA and Adipoq-Cx43 mice (Figure 4D). Lastly, we performed targeted cytokine measurement and found that IL-15 was significantly suppressed in Adipoq-Cx43 serum (Figure 4E). However, no literature implicates IL-15 in food-intake regulation.

### 3.5 Serum GDF15 was elevated but did not mediate food intake in Adipoq-Cx43 mice

**Figure 5:**
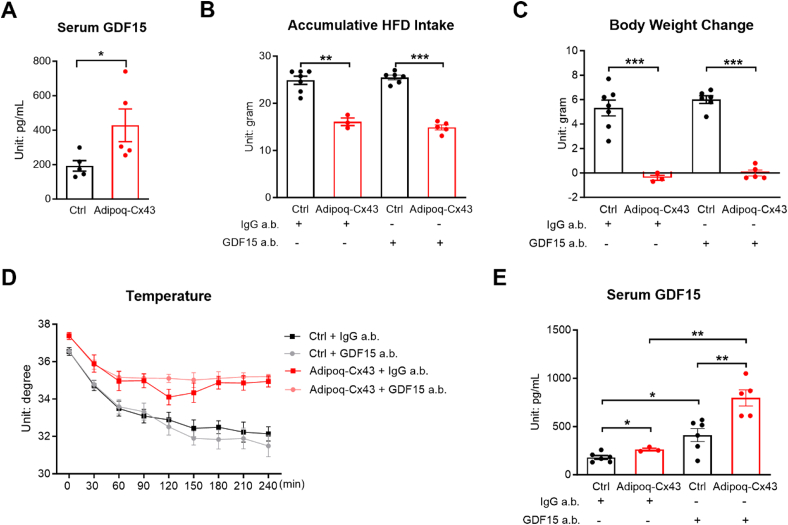
Serum GDF15 was elevated but did not mediate food intake in Adipoq-Cx43 mice. (A) Serum GDF15 of control and Adipoq-Cx43 mice treated with Dox200 HFD for 3 weeks (n = 5 mice per group). (B) Accumulative HFD intake and (C) body weight change of control and Adipoq-Cx43 mice on HFD over 9 days when treated with IgG isotope control or GDF15 antibody (n = 7 control mice for IgG isotope control antibody; n = 3 Adipoq-Cx43 mice for IgG isotope control antibody; n = 6 control mice for GDF15 antibody; n = 5 Adipoq-Cx43 mice for GDF15 antibody). (D) The body temperature of control and Adipoq-Cx43 mice upon 4-hour cold exposure (n = 9 control mice for IgG isotope control antibody; n = 5 Adipoq-Cx43 mice for IgG isotope control antibody; n = 8 control mice for GDF15 antibody; n = 6 Adipoq-Cx43 mice for GDF15 antibody). (E) Serum GDF15 of control and Adipoq-Cx43 mice after antibody injection; the mice were from Panel (B); one outlier was excluded from the control IgG isotope group. For Panel (A), comparisons between genotypes were performed using unpaired two-tailed *t*-tests; two-way ANOVA followed by post-hoc Sidak multiple comparisons were used for Panels (B), (C), and (E); in Panel (D), time point–specific comparisons between genotypes were analyzed using serial *t-*tests. All data are mean ± SEM. ∗∗∗P < 0.001, ∗∗P < 0.01, and ∗P < 0.05.

Another gene that was significantly upregulated in the Adipoq-Cx43 iWAT RNA-seq dataset is *Gdf15*, which encodes growth differentiation factor 15 (GDF15), a known secreted protein promoting anorexia [26]. Three weeks of HFD treatment significantly increased serum GDF15 levels in Adipoq-Cx43 mice (Figure 5A), suggesting that enhanced adipose tissue *Gdf15* expression translated to more protein and increased secretion of the GDF15 protein from the adipose tissue to circulation. To determine whether GDF15 mediates food intake suppression in Adipoq-Cx43 mice, a GDF15-neutralization antibody was used at a dose previously shown to be sufficient to neutralize LPS-induced GDF15 surge (>2 ng/mL), which is markedly higher than the GDF15 level observed (∼400 pg/mL) in Adipoq-Cx43 mice [27]. The GDF15-neutralizing antibody did not affect food intake or body weight for Adipoq-Cx43 mice on HFD (Figure 5B, C). GDF15-neutralizing antibody did not affect cold resistance of Adipoq-Cx43 mice either (Figure 5D). Notably, GDF15-neutralization antibody significantly increased serum GDF15 (Figure 5E)—functionally blocking GDF15-elicited signaling and thus causing a compensatory synthesis of GDF15, a common phenotype seen in scenarios of inhibiting a serum signaling factor. Technically, these GDF15 are still detectable by ELISA due to a different binding antibody used in the ELISA assay.

### 3.6 Adipose tissue sensory neurons are implicated in adipocyte Cx43's effect on food intake

**Figure 6:**
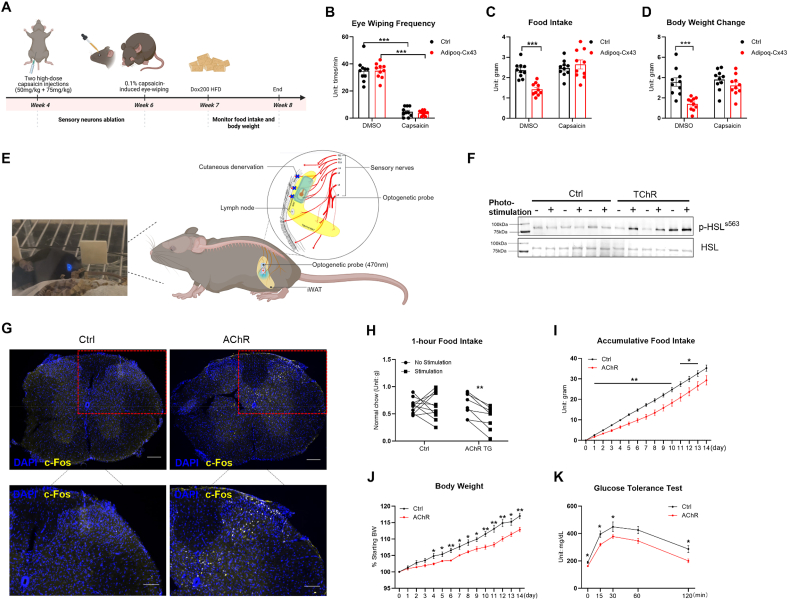
Ablation of sensory neurons abolishes adipocyte Cx43's effect on food intake, and optogenetic activation of sensory neurons in adipose tissue is sufficient to suppress food intake. (A) Schematic diagram of capsaicin-induced sensory ablation in control and Adipoq-Cx43 mice. (B) Number of eye-wiping events of control and Adipoq-Cx43 mice in 1 min upon application of low-dose capsaicin eye drops. (C) Food intake of control and Adipoq-Cx43 mice with vehicle or high-dose capsaicin injection. (D) One-week bodyweight changes in control and Adipoq-Cx43 mice after vehicle or capsaicin injection (n = 10 mice for each group in Panels (B)–(D)). (E) Photo of an implanted LED probe with blue light in the wired cage and a schematic diagram illustrating the surgical anatomy and position of the LED probe in iWAT. (F) p-HSL^s563^ and HSL protein levels in iWAT from control and TChR mice with photostimulation on one side and without photostimulation on the counter-lateral side. (G) Representative c-Fos staining in spinal cords at the T10 level of control and AChR mice with 1-hour stimulation; scale bar: upper panel 100 μm, lower panel 50 μm. (H) One-hour food intake of control and AChR mice with or without photostimulation (n = 11 mice for the control group; n = 8 for the AChR group). (I) Accumulative food intake and (J) body weight progression in control and AChR mice or Advillin-Cre control mice with bilateral probe implantation and subjected to daily stimulation at Zeitgeber time (ZT) 11 for 1 h (n = 6 mice for each group). (K) GTT in control and AChR mice with daily 1-hour photostimulation and 2-week HFD treatment (n = 6 mice for each group). For Panels (B)–(D), two-way ANOVA followed by post-hoc Sidak multiple comparisons were used; a paired *t*-test was used in Panel (H); time point–specific comparisons between genotypes were analyzed using serial *t*-tests for Panels (I)–(K). All data are mean ± SEM. ∗∗∗P < 0.001, ∗∗P < 0.01, and ∗P < 0.05.

After failing to identify secreted proteins in Adipoq-Cx43 mice mediating adipocytes to central regulation of food intake, we suspect that adipose tissue afferent sensory neurons may play a role in this process. We first used high-dose capsaicin (50 mg/kg on the first day followed by 75 mg/kg on the second day, subcutaneous injection) to ablate sensory neurons systemically in young mice (Figure 6A). The efficacy of ablation was confirmed by the reduction in a 0.1% capsaicin-induced eye-wiping behavior, a commonly used reflexive assay for sensory neuron function (Figure 6B). At the age of 7 weeks, mice were switched to Dox200 HFD. Sensory neuron ablation effectively abolished the reduced food intake observed in Adipoq-Cx43 mice on Dox200 HFD over the course of 1 week (Figure 6C) and restored their body weight gain to a comparable level of Adipoq-rtTA control mice (Figure 6D). Next, we employed optogenetics to activate sensory neurons specifically in the iWAT depots to test whether activation of sensory neurons could suppress food intake as Cx43 overexpression did. The wireless optogenetic system was first validated using the well-established TH-Cre:LSL-ChR2 (TChR) mice, in which ChR2 is expressed in tyrosine hydroxylase–positive sympathetic neurons, leading to increased HSL phosphorylation in iWAT upon light stimulation (Figure 6E, F, and Fig. S3). To target sensory neurons, we used Advillin-Cre:LSL-ChR2 (AChR) mice, where ChR2 is expressed in peripheral sensory neurons under the Advillin promoter. Light stimulation of iWAT in AChR mice induced c-Fos expression in the ipsilateral dorsal horn of the spinal cord (T12–L2), confirming successful activation of iWAT-innervating sensory afferents (Figure 6G and Fig. S4). Within 1 h of stimulation, the intake of LFD was suppressed by 37% in AChR mice when compared to the food intake measured without photoactivation (Figure 6H). In contrast, photoactivation had no effect on LSL-ChR2 (control) mice implanted with the functioning LED probe (Figure 6H), excluding the possibility that light, heat from the probe, or the surgical procedure contributed to the changes in food intake. To assess whether activation of iWAT-innervating sensory neurons could exert beneficial metabolic effects as observed in adipocyte-specific Cx43 overexpressing mice, we applied daily stimulation (1 h per session before dark cycle) for 2 weeks, which significantly reduced cumulative food intake (Figure 6I) and body weight gain on HFD (Figure 6J), leading to improved glucose tolerance (Figure 6K). All these data demonstrate that activation of iWAT sensory neurons recapitulates the effects of adipose tissue Cx43 overexpression on food intake and related glucose homeostasis.

### 3.7 Adipocytes communicate to sensory neurons via gap junctions to modulate sensory neurons' electrical firing activity

**Figure 7:**
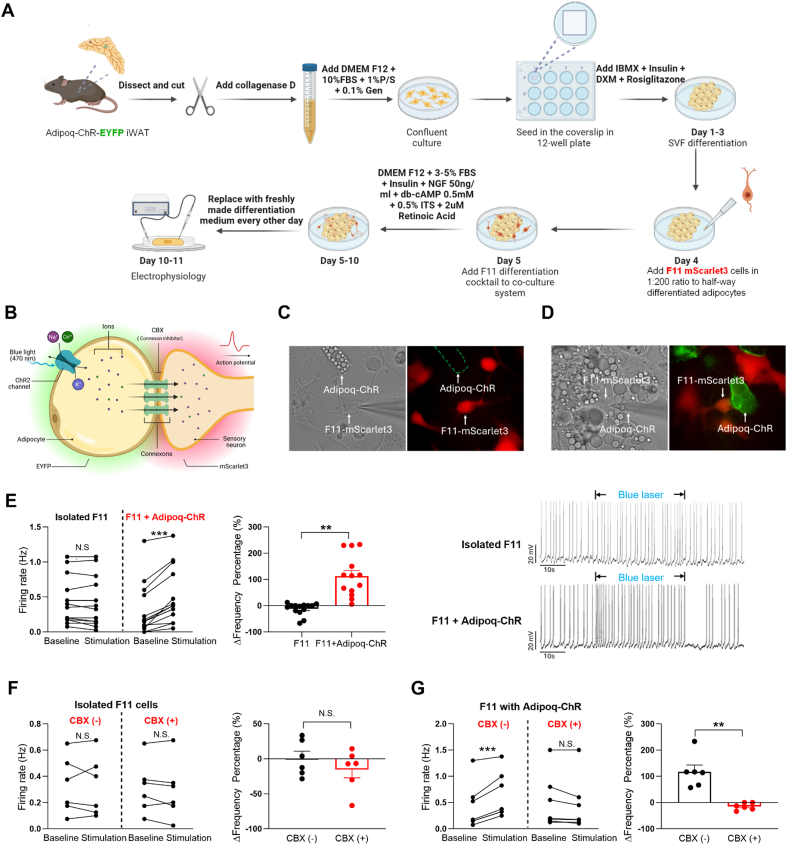
Adipocytes pass electrical signals to sensory neurons via gap junctions to modulate sensory neurons' electrical firing. (A) Schematic flowchart illustrating the co-culture of primary SVF with the sensory neuron cell line F11. Details are included in the Methods section. IBMX: 3-isobutyl-1-methylxanthine; DXM: dexamethasone. (B) Diagram depicting the electrical synapses between adipocytes and neurons. (C) Representative image of isolated F11 cells. (D) Representative image of F11 cells in contact with adipocytes from Adipoq-ChR mice. (E) Firing frequency of isolated F11 cells and F11 cells in contact with differentiated Adipoq-ChR adipocytes, with or without blue laser stimulation. The middle panel shows the frequency difference before and after stimulation; the right panel presents a representative electrophysiological recording (n = 13 cells recorded for the isolated F11 cells; n = 14 for the F11 cells in contact with differentiated adipocytes). (F) Firing frequency of isolated F11 cells with or without carbenoxolone (CBX) treatment. The bar graph on the right shows the frequency difference before and after stimulation (n = 6 for each group). (G) Firing frequency of F11 cells in contact with differentiated Adipoq-ChR adipocytes with or without CBX treatment. The bar graph on the right shows the frequency difference before and after stimulation (n = 6 for each group). For Panels (E)–(G), a paired *t*-test was used on the left, and an unpaired *t*-test was used on the right. All data are mean ± SEM. N.S.: not significant. ∗∗∗P < 0.001, ∗∗P < 0.01, and ∗P < 0.05.

After finding that adipose tissue sensory neurons are involved in the effects of adipocyte Cx43 on food intake, we sought to understand how adipocytes activate sensory neurons. Since studying adipocyte–sensory neuron communication in vivo is challenging, we aimed to model this interaction in vitro using co-culture. We first tested the OMEGA^ACE^ triple-chamber neuronal co-culture device, but few F11 neurites extended through the microchannels, and none contacted mature adipocytes on the other side. We then developed a simpler co-culture system by directly mixing both cell types in a single dish. Adipocytes were differentiated from the stromal vascular fraction (SVF) of Adipoq-Cre:LSL-ChR2-EYFP (Adipoq-ChR) mice and expressed EYFP; F11 sensory neurons were transfected with an mScarlet3 construct, allowing us to visualize them in red (Figure 7A, B). A major technical hurdle was that serum is needed for adipocyte differentiation but inhibits F11 differentiation, while db-cAMP promotes F11 differentiation but also causes lipolysis and adipocyte death. We optimized the serum and db-cAMP concentrations by testing different combinations. Adipocytes were first differentiated, and F11 cells were added on Day 4 (Figure 7A). Then, electrophysiology was employed to record the neuronal electrical activity of isolated F11 cells (Figure 7C) or F11 cells in contact with photoactivated adipocytes (Figure 7D), identified by their spatial positioning under a fluorescent microscope. Upon activation of channelrhodopsins in the adipocytes through use of the blue laser, F11 cells coupled with adipocytes exhibited an increase in firing frequency, while isolated F11 cells showed no change (Figure 7E and Fig. S5A and S5B). This adipocyte-mediated increase in sensory neuron firing was completely blocked by a gap junction inhibitor, carbenoxolone (CBX), while treating isolated sensory neuronal cells with CBX did not affect firing frequency (Figure 7F,G, and Fig. S5C and S5D). Altogether, these findings provide evidence that gap junctions formed between adipocytes and sensory neurons facilitate the transmission of ion currents from adipocytes to F11 sensory neurons, affecting their electrical firing activity.

## 4 Discussion

We started with an observation that adipose tissue–specific expression of gap junction protein Connexin43 modulates food intake. After a strenuous failed search for adipose tissue–secreted appetite-regulating factors, we found that sensory neurons were required for the regulation of food intake in our Adipoq-Cx43 mice. On the other hand, activation of sensory neurons in iWAT using wireless optogenetics was sufficient to suppress food intake. Via the co-culture system, we presented strong evidence that adipocytes and sensory neuronal cells formed gap junctions, allowing adipocytes to modulate sensory neuronal electrical firing. All these data suggest the existence of electrical synapses between adipocytes and sensory neurons to communicate with the brain to regulate food intake, a high-level central behavior.

Electrical synapses, formed by gap junctions, are physical connections between neurons or between neurons and other types of cells; such synapses allow rapid, bidirectional flow of electrical current [28]. Electrical synapses represent an ancient form of neural communication, crucial in early evolution for rapid signaling and retained in modern nervous systems for specialized functions, e.g., retinal amacrine cells and inferior olive neurons [28]. During development, electrical synapses help establish early neural networks before being replaced by chemical synapses in most circuits [29].

No previous work has investigated how adipose tissue sensory neurons modulate a central nervous system–governed behavior (e.g., food intake). However, hints from how adipose tissue sensory neurons affect the systemic sympathetic system have implicated such a regulation. Sympathetic activation typically acts as an inhibitory signal to suppress food intake. In one report, activation of sensory nerves by low-dose capsaicin microinjections to iWAT of rats enhanced renal sympathetic nerve activity and mean arterial pressure in the kidneys, suggesting the involvement of WAT-specific sensory afferents in activating sympathetic output to other organs [30,31]. Injection of leptin into laboratory rat eWAT provokes an increase in the firing rate of sensory nerves emanating from the depot and an increase in sympathetic nerve activity in the contralateral eWAT pad [32,33]. Furthermore, another study showed that sensory denervation of iWAT reduces sympathetic tone to BAT [34]. Altogether, iWAT sensory activation positively regulates the systemic sympathetic system and thus is expected to inhibit food intake.

However, in the WAT depot proper, the functional relationship between sensory and sympathetic nerves remains debated [5]. Through a combination of viral tracing and denervation in iWAT, it has been shown that iWAT sensory neurons function to inhibit sympathetic neuronal activity [35]. In contrast, other studies using iWAT sensory denervation by high-dose capsaicin showed reduced sympathetic output in iWAT 24 h after cold exposure [34], demonstrating that sensory nerve activation in iWAT is required for sympathetic activity in the same depot.

The apparent ease of regulating food intake by many mechanisms conceals the complexity of the underlying decision process. Eating or not is an outcome resulting from processing visual and olfactory cues, with cognitive input determining safety and benefits, as well as integrating various subconscious regulations [36]. Research in mice has found that appetite can be affected (mostly suppressed) by vast amounts of biological processes [37]. Then, the question is the relative importance of this appetite regulation by adipose tissue sensory neurons compared to other mechanisms. It is obviously not a strong and direct regulator like AgRP neurons in the hypothalamus region [38,39] or the more recently identified subcortical feeding circuit that controls jaw movement [40], as we did not notice an immediate change in eating behavior following opto-stimulation of sensory neurons in iWAT. Here, aside from classical homeostatic and hedonic eating classification, we propose a two-layer appetite-regulating model: there is a basal constant-on signal that drives eating whenever food is available, as this is expected to provide evolutionary advantages; on top of the foundation layer, many other neuronal mechanisms, with various levels of priority, function to stop eating, which forms the regulatory layer. Those regulatory mechanisms can originate from the gastrointestinal (GI) tract to relay the mechanical stress in the GI tract to the brain to stop eating to prevent the rupture of the GI tract; the mechanisms could also be simply a neuronal response to danger, governing fight–or–flight reaction when survival is a higher priority than eating; in our current society, self-awareness and self-perception of beauty could also play a significant role in suppressing eating behavior. The signaling from adipose tissue sensory neurons also serves as part of this modulating layer, possibly surveilling the state of the adipose tissue to prevent excessive fat accumulation. This mechanism, operating in the background in the modulatory layer, appears to have a relatively low priority. However, activating the mechanism, by design, is expected to have the advantage of being very safe.

In addition to reduced food intake, Adipoq-Cx43 mice exhibited lower RER and decreased energy expenditure, yet paradoxically maintained higher body temperature and improved cold defense under chronic cold exposure. At first glance, the reductions in RER and energy expenditure could be interpreted as secondary to reduced food intake; however, on normal chow, the decline in RER precedes any significant change in intake, suggesting a primary effect of adipose Cx43 on substrate utilization. This effect cannot be explained by differences in lipolysis, nor by the significant downregulation of several lipolytic genes. The precise mechanism by which adipocyte Cx43 lowers RER and whether it contributes to protection against HFD-induced weight gain remains to be investigated. Interestingly, Adipoq-Cx43 mice display reduced adiposity but maintain body temperature more effectively, indicating that adipose tissue quality may be more important than quantity in thermoregulation. This observation also rules out a lipodystrophic phenotype, since lipodystrophic mice typically show impaired thermoregulation under cold stress [41]. In mice, body temperature is governed by an intrinsic “set point,” rather than simply by heat generation, insulation, or fur density [41]. It will be of great interest to understand how adipose tissue Cx43 could contribute to this set point.

In summary, adipose sensory nerves have long been suspected to detect changes in adipocyte released lipids like arachidonic acid and eicosapentaenoic acid to communicate changes in the local lipid milieu to the brain [42]. Our optogenetic and co-culture experiments strongly support an alternative mechanism, demonstrating that adipocytes communicate with sensory neurons via electrical synapses within the depot to influence central neuronal function. Future studies will aim to elucidate the ultrastructure and connexin composition of these electrical synapses, identify the initiating signals that activate adipocytes and sensory neurons, map the central projections of specific sensory neuron populations, and explore potential clinical applications—for example, using the Rec2 AAV serotype [43] to deliver Gja1 to adipose tissue.

Our manuscript also has several limitations. Capsaicin-induced sensory ablation is not specific to sensory neurons in iWAT. More targeted approaches—such as local capsaicin injection or delivery of an AAV expressing Cre-dependent diphtheria toxin subunit A into the fat pad of Advillin-Cre mice—would provide greater depot-specific sensory neuron targeting. While the appetite phenotype is a direct consequence of adipocyte Cx43 expression, we currently lack direct evidence that Cx43, rather than other connexin isoforms, is responsible for the gap junction–mediated electrical communication between adipocytes and sensory neurons. It is, in fact, quite possible that additional connexins also contribute to this physiology, a hypothesis that would require genetic approaches targeting specific connexin isoforms for confirmation.

## CRediT authorship contribution statement

**Xi Chen:** Writing – review & editing, Writing – original draft, Visualization, Validation, Methodology, Investigation, Formal analysis. **Xing Fang:** Writing – review & editing, Methodology, Investigation, Funding acquisition, Formal analysis. **Hong Zhou:** Writing – review & editing, Visualization, Investigation, Formal analysis. **Jieyi Meng:** Writing – review & editing, Investigation. **Yang He:** Writing – review & editing, Methodology. **Leon G. Straub:** Writing – review & editing, Methodology. **Andrew Lemoff:** Writing – review & editing, Investigation. **Clair Crewe:** Writing – review & editing, Methodology. **Shangang Zhao:** Writing – review & editing, Funding acquisition. **Yong Xu:** Writing – review & editing, Resources, Methodology. **Yi Zhu:** Writing – review & editing, Writing – original draft, Visualization, Supervision, Resources, Project administration, Funding acquisition, Conceptualization.

## Fundings

This work was supported by 10.13039/100000002NIH R01DK136619, R01DK136532, and 10.13039/100000199USDA/ARS (CRIS 58-3092-5-008) to Y. Z.; the NIH R00AG068239, R01DK138035, and R01AG084646 to S. Z.; and 10.13039/100000062NIDDK 1F32DK 138685-01A1 to X.F.

## Declaration of competing interest

The authors declare no conflicts of interest.

## Data Availability

Data will be made available on request.
