# Supplementary material for: Regulation of food intake by Connexin43 via adipocyte-sensory neuron electrical synapses
Source: Mol Metab. 2025 Sep 5;101:102247. doi: 10.1016/j.molmet.2025.102247 (PMC12544212; doi:10.1016/j.molmet.2025.102247)
Supplement: Multimedia component 2 [file mmc2.docx]

**Regulation of food intake by Connexin43 via adipocyte-sensory neuron electrical synapses**

Xi Chen^1,#^, Xing Fang^1,#^, Hong Zhou^1^, Jieyi Meng^1^, Yang He^2^, Leon G. Straub^3^, Andrew Lemoff^4^, Clair Crewe^5^, Shangang Zhao^6^, Yong Xu^1^, Yi Zhu^1,*^

^1^ Children's Nutrition Research Center, Department of Pediatrics, Baylor College of Medicine, Houston, TX, 77030, USA

^2^ Jan and Dan Duncan Neurological Research Institute at Texas Children's Hospital, Baylor College of Medicine, Houston, 77030, USA

^3^ Department of Biochemistry and Molecular Cell Biology, University Medical Center Hamburg-Eppendorf, Martinistr. 52, 20246, Hamburg, Germany

^4^ Department of Biochemistry, The University of Texas Southwestern Medical Center at Dallas, Dallas, TX, 75390, USA

^5^ Department of Cell Biology and Physiology, Washington University School of Medicine, St. Louis, MO, USA

^6^ Barshop Institute for Longevity and Aging Studies, Division of Endocrinology, Department of Medicine, University of Texas Health Science Center at San Antonio, San Antonio, TX, USA

**Supplementary Figure 1**

**Figure S1: (A) Expression of *Gja1* in iWAT from control and** Adipoq-Cx43 mice treated with 7 days of Dox200 HFD (n = 8 mice for control, n = 9 mice for Adipoq-Cx43). (B) Protein levels of Cx43 in the iWAT of Adipoq-Cx43 mice and control mice treated with Dox200 LFD for 1 week.


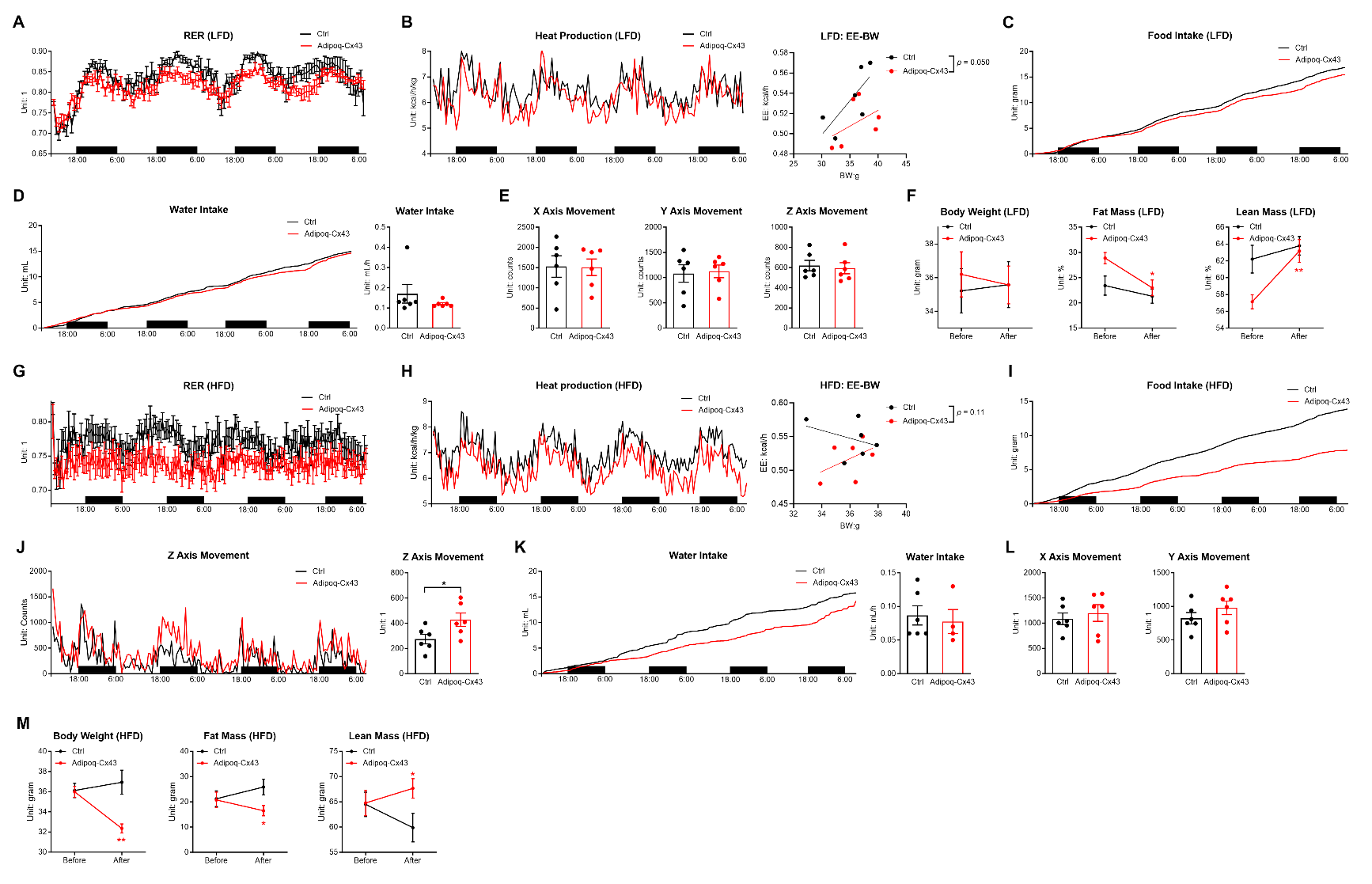
**Supplementary Figure 2**

**Figure S2:** (A) RER, (B) heat production (with ANCOVA analysis shown on the right), (C) food intake, (D) water intake, and (E) three-axis activities of control and Adipoq-Cx43 mice treated with Dox200 LFD. (F) The changes of body weights and body compositions of control and Adipoq-Cx43 mice on Dox 200 LFD before and after the cage study (for Panels (A)–(F), n = 6 mice per group). (G) RER, (H) heat production (with ANCOVA analysis shown on the right), (I) food intake, (J) activities in Z axis, (K) water intake, and (L) activities in X and Y axis of control and Adipoq-Cx43 mice treated with Dox200 HFD. (M) The changes of body weights and body compositions of control and Adipoq-Cx43 mice on Dox200 HFD before and after the cage study (for Panels G–M, n = 6 mice per group; 2 mice in the Adipoq-Cx43 group were excluded from water intake analysis due to leakage from the water bottle). Two-way ANOVA was used for line graphs in Panels (A)–(D) and (G)–(K); unpaired two-tailed *t*-tests were used to compare different genotypes for all bar graphs and Panel (M). All data are mean ± SEM. ***P* < 0.01, **P* < 0.05.

**Supplementary Figure 3**


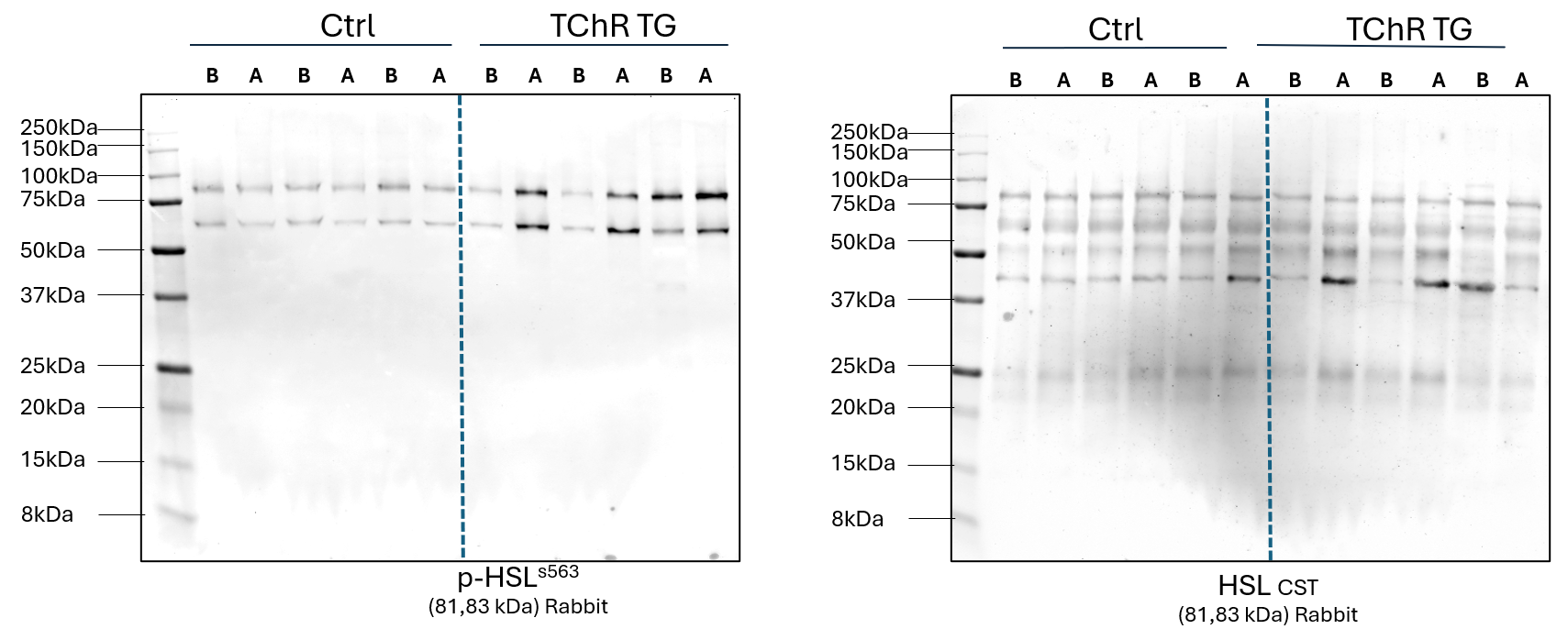


**Figure S3:** Protein levels of p-HSL^s563^ and HSL in the iWAT of control and TChR TG mice with or without photo-stimulation. B: before photo-stimulation; A: after photo-stimulation.


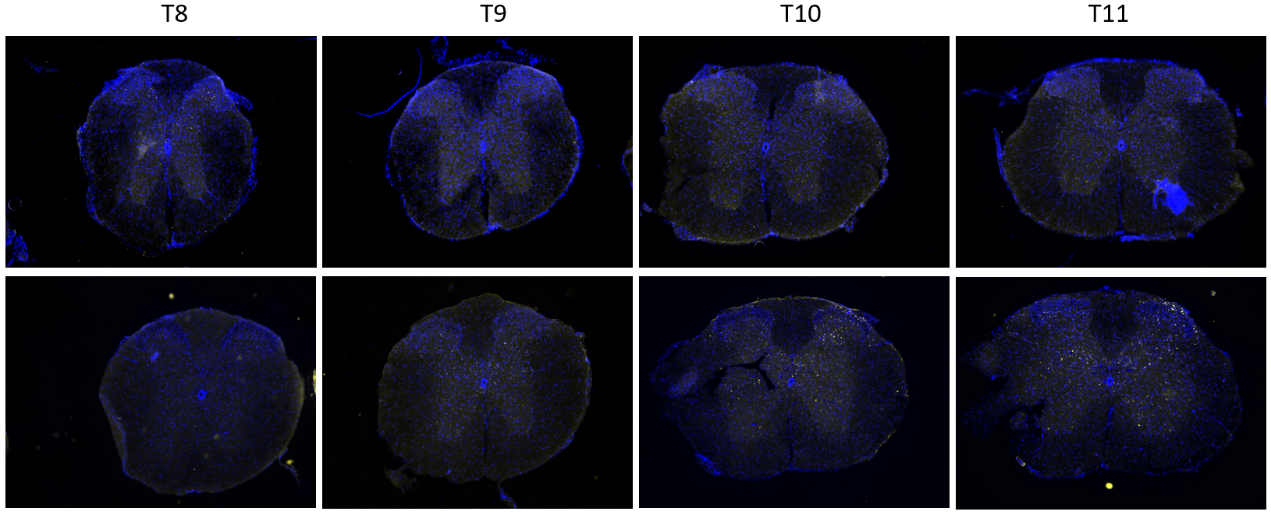
**Supplementary Figure 4**

AChR

Control


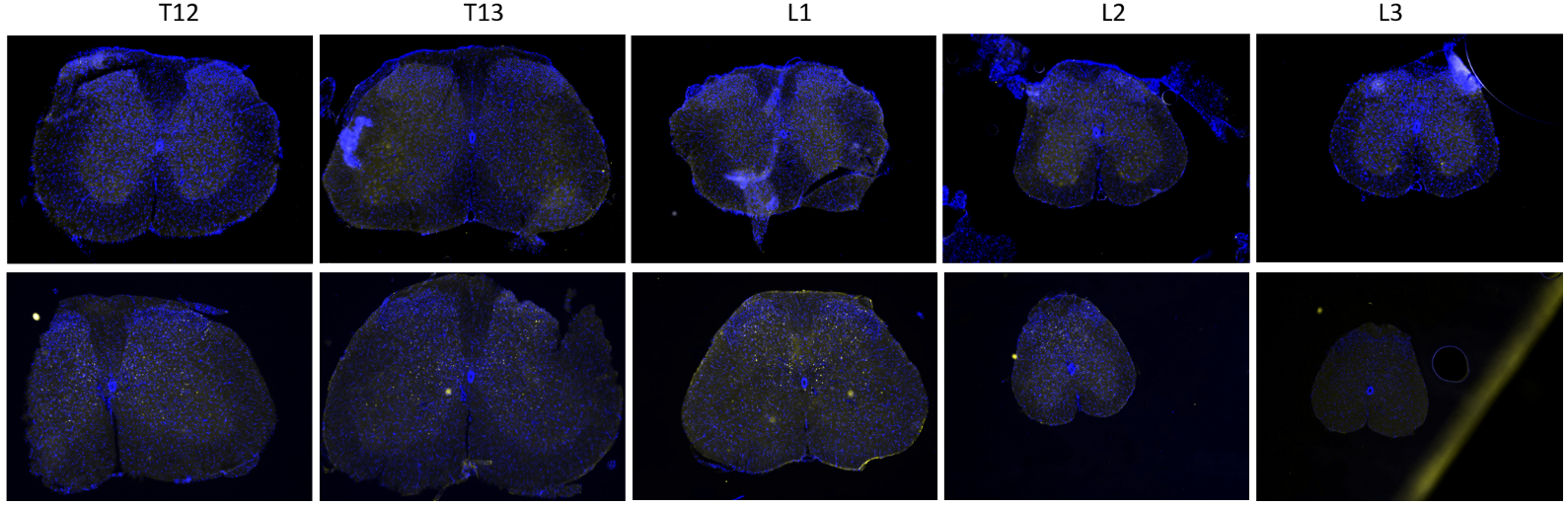


AChR

Control

**Figure S4:** Representative c-Fos staining in spinal cords at T8-L3 levels of control and AChR mice with one-hour stimulation. Blue: DAPI; white: c-Fos.

**Supplementary Figure 5**

**(A**) **Isolated F11 cells:**


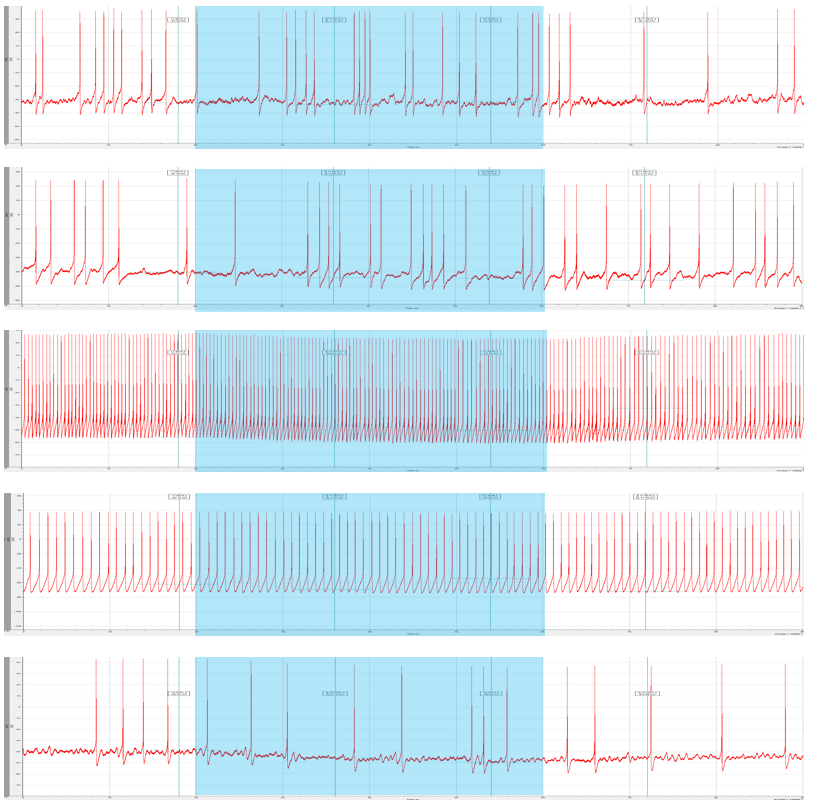

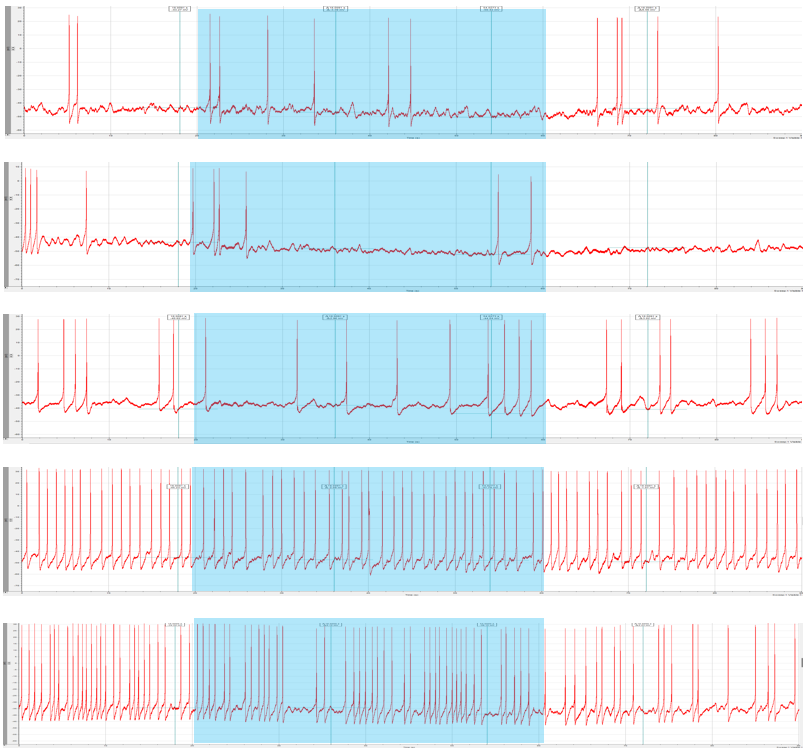

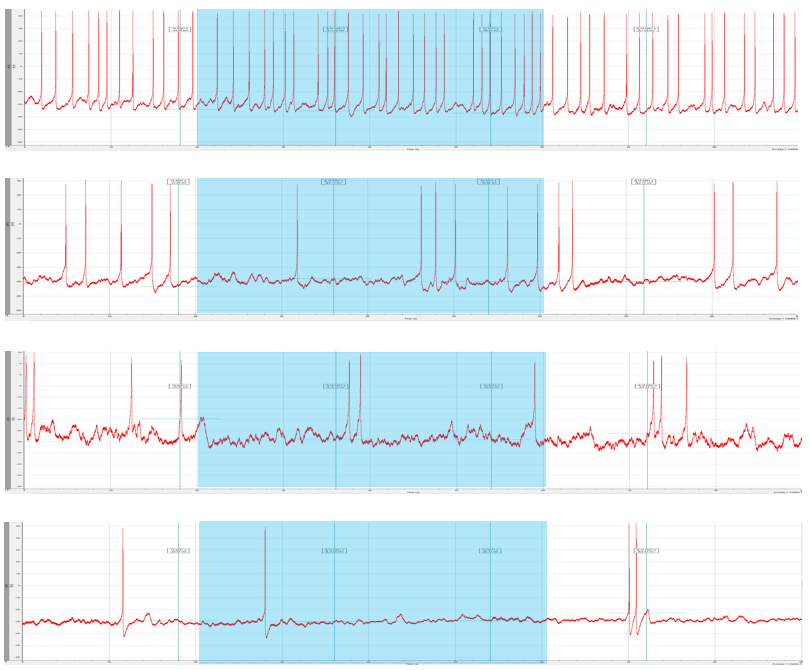


No stimulation

Blue light stimulation 20 Hz


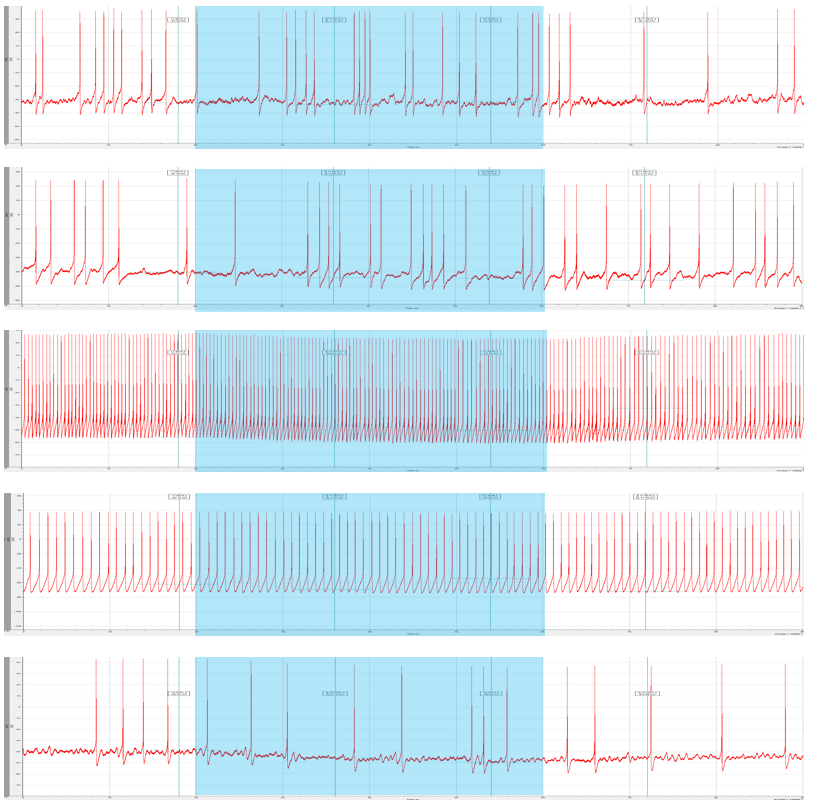


**(B) F11 + Adipoq-ChR:**


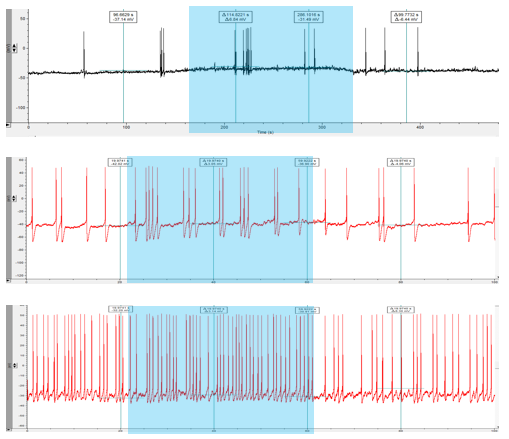


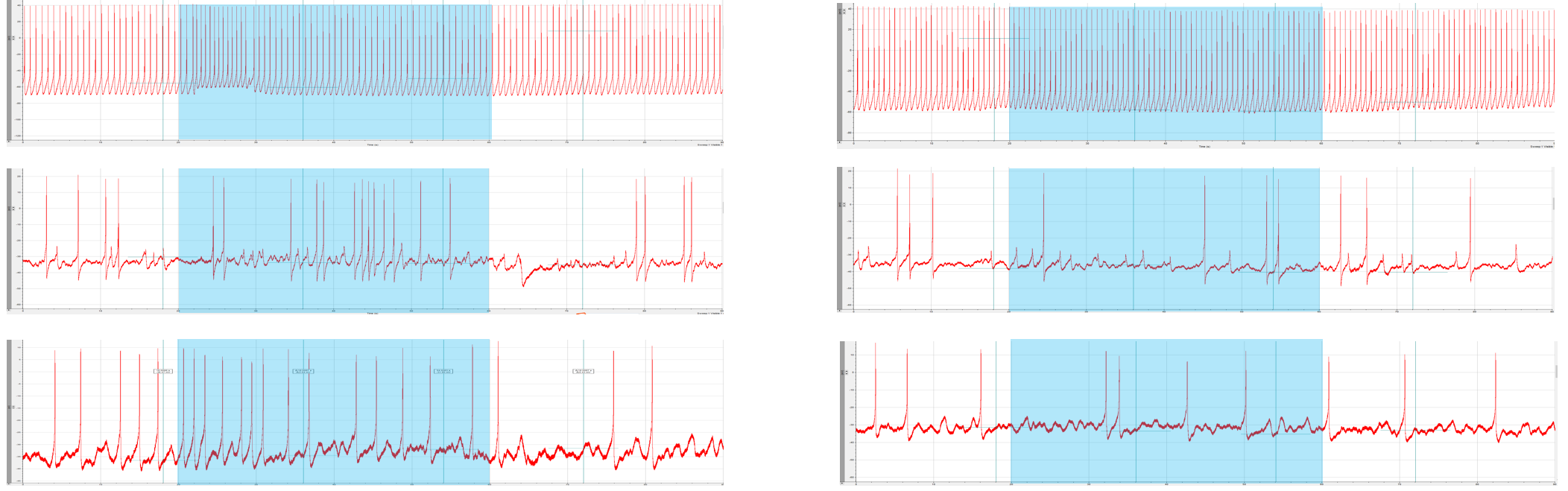

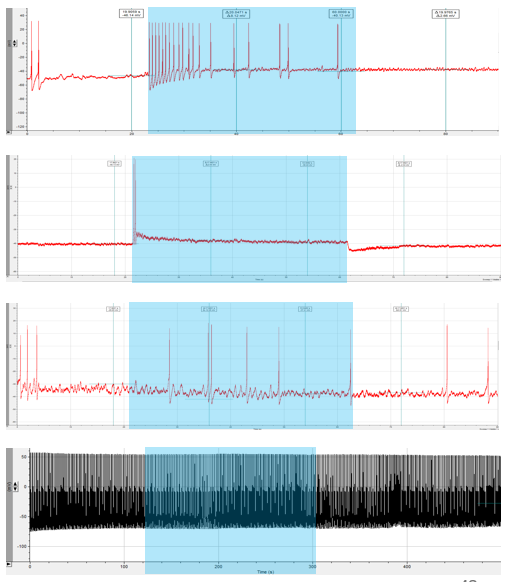


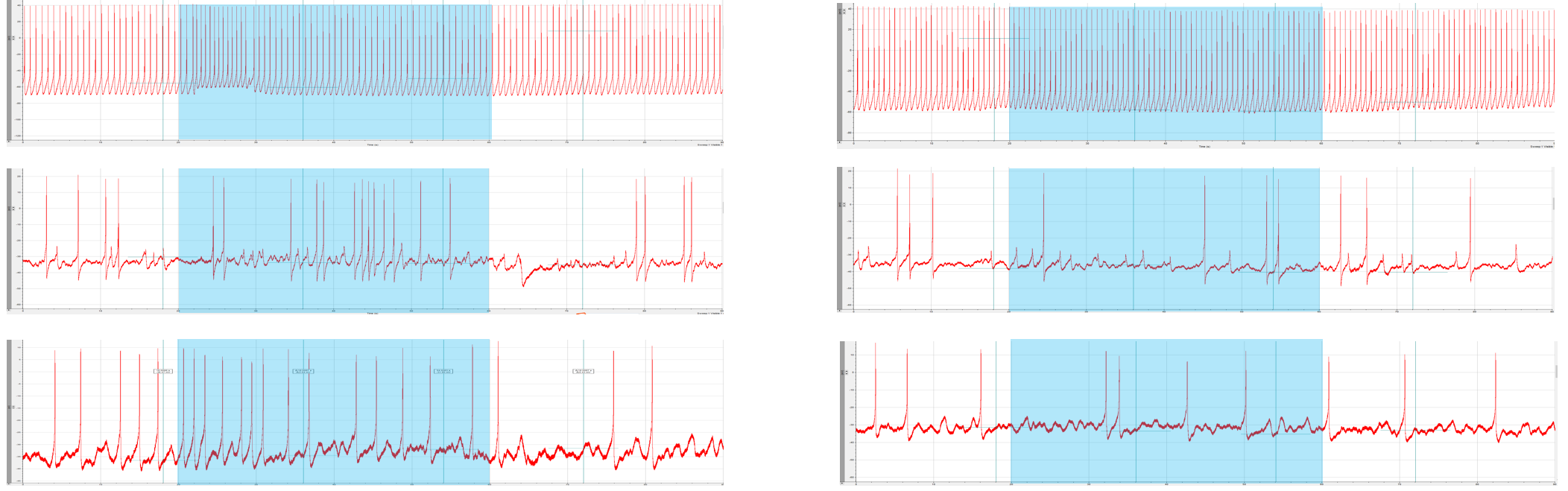


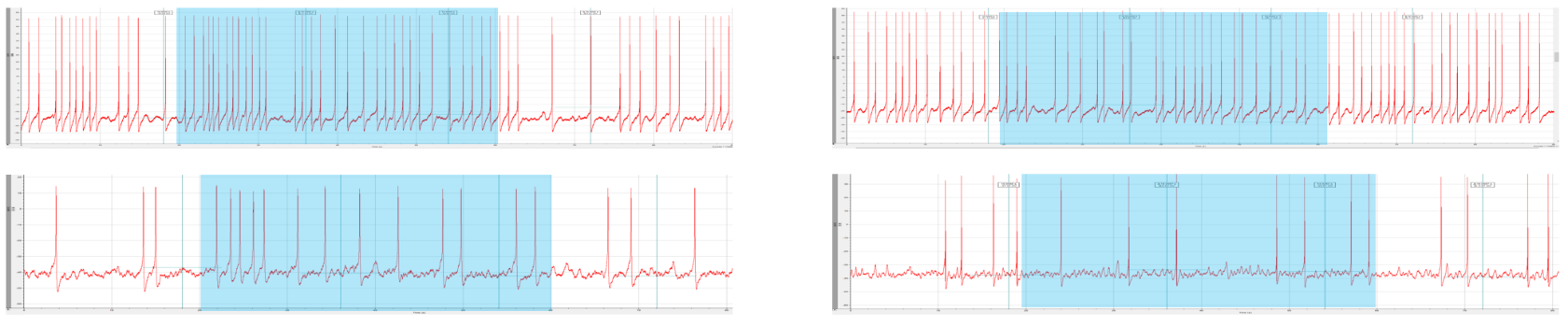

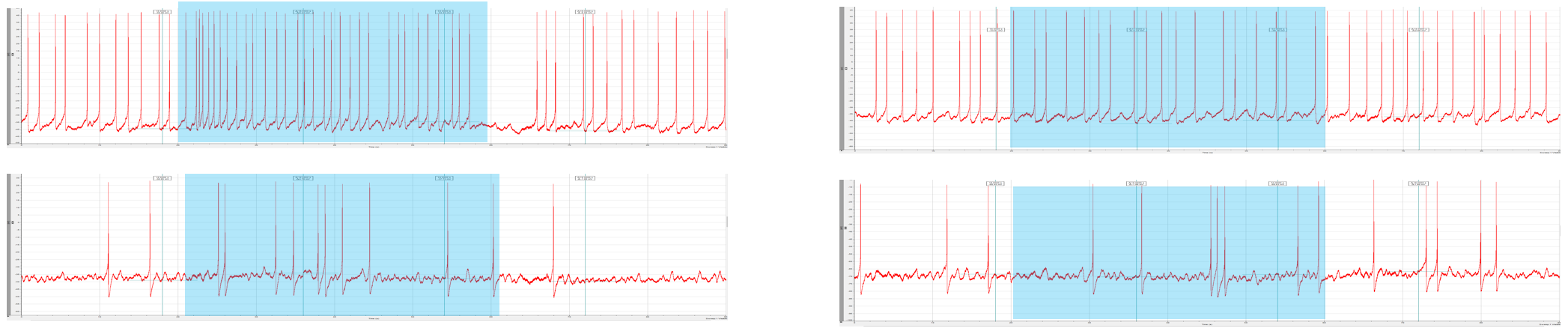


**(C**) **Isolated F11 cells treated with CBX (3 μL, 10 mM, 3 minutes):**


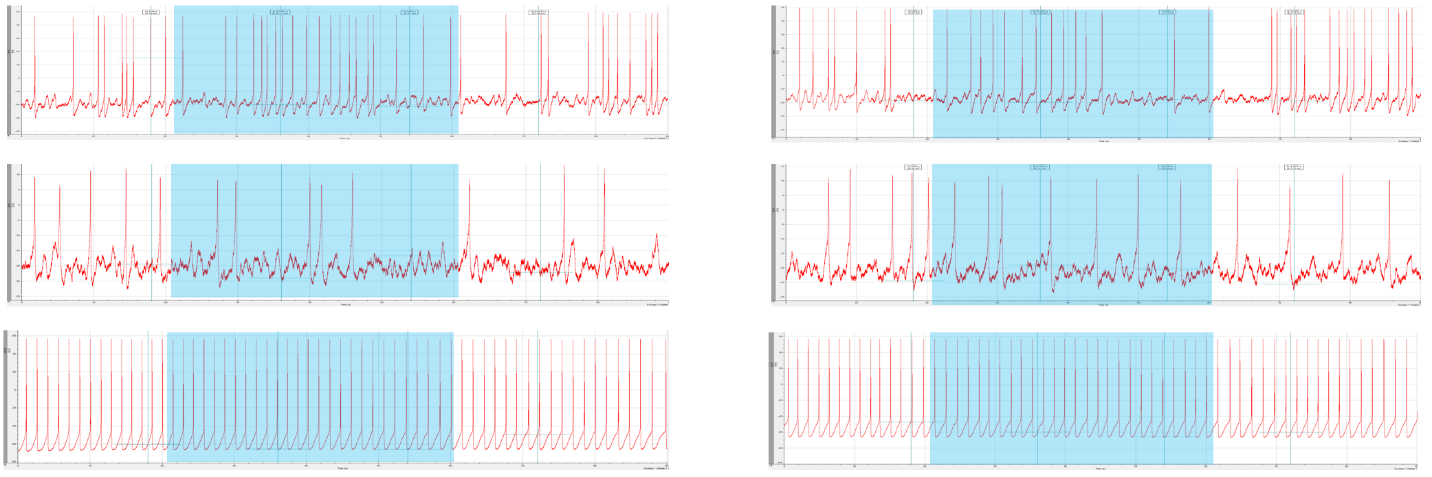
 Before CBX After CBX


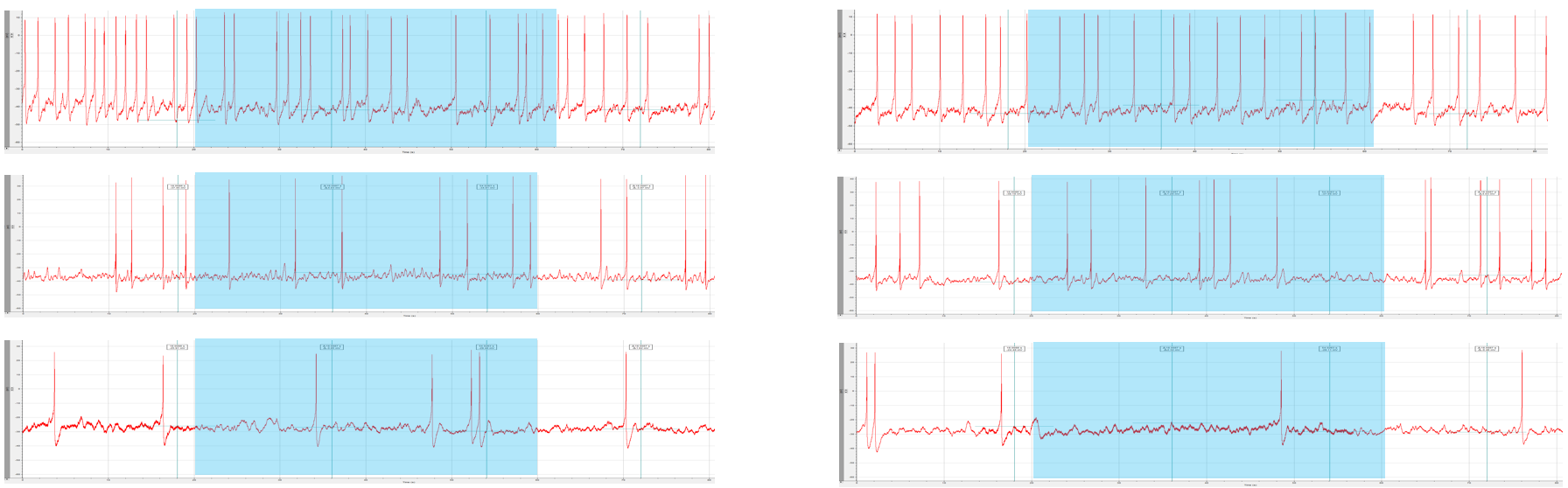


**(D) F11 + Adipoq-ChR treated with CBX (3 μL, 10 mM, 3 minutes):**

Before CBX After CBX


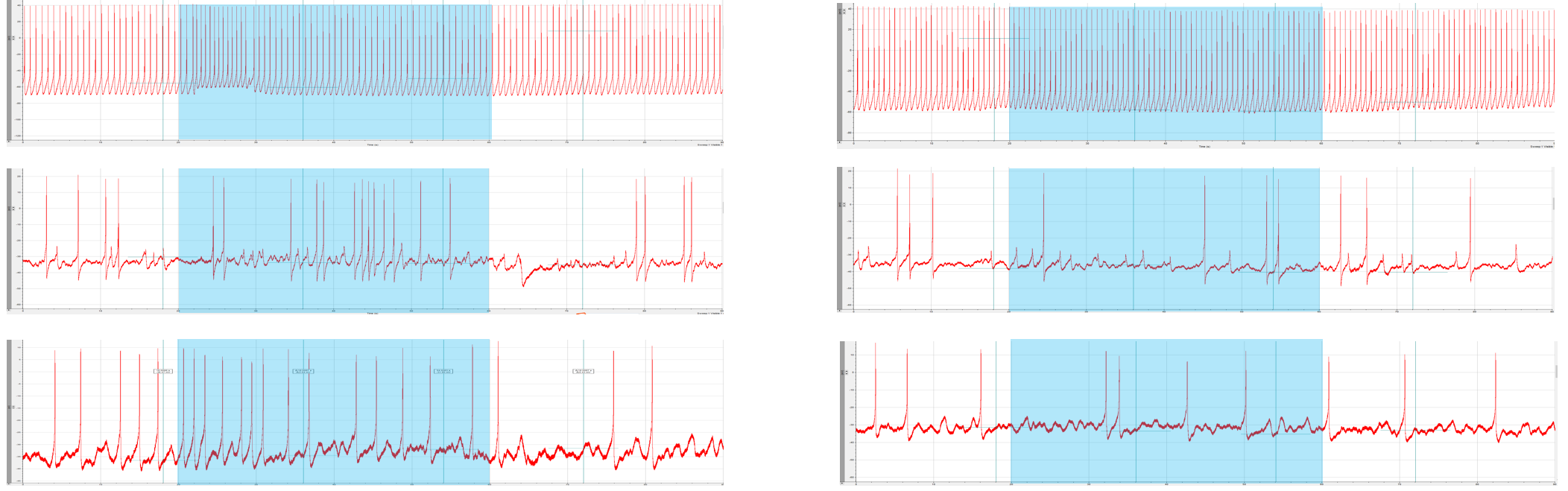


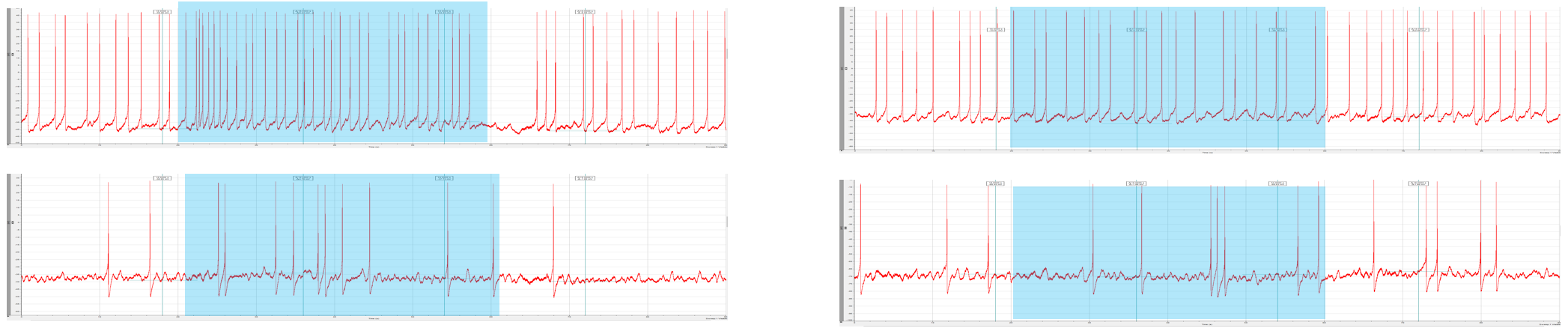


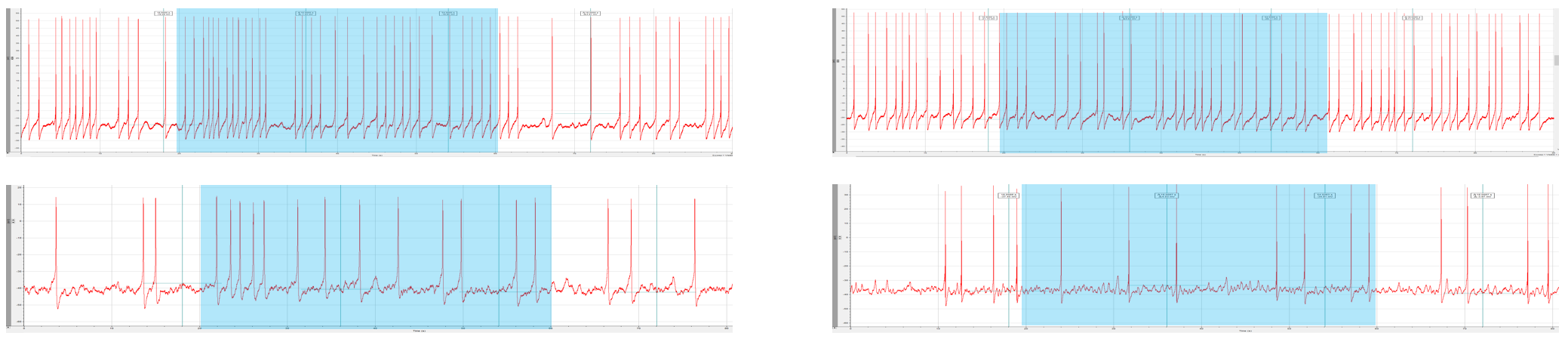


**Figure S5.** Electrophysiological recording of (A) isolated F11 cells and (B) F11 cells in contact with differentiated adipocytes (n = 13 cells recorded for the isolated F11 cells and n = 14 for the F11 cells in contact with differentiated adipocytes). (C) Electrophysiological recording of isolated F11 cells with or without CBX treatment, before and after stimulation (n = 6). (D) Electrophysiological recording of F11 cells in contact with differentiated adipocytes from Adipoq-ChR mice, before and after with or without CBX treatment (n = 7).
